# A system pharmacology Boolean network model for the TLR4-mediated inflammatory response in early sepsis

**DOI:** 10.1007/s10928-022-09828-6

**Published:** 2022-10-19

**Authors:** Feiyan Liu, Linda B. S. Aulin, Sebastiaan S. A. Kossen, Julius Cathalina, Marlotte Bremmer, Amanda C. Foks, Piet H. van der Graaf, Matthijs Moerland, Johan G. C. van Hasselt

**Affiliations:** 1 Leiden Academic Centre for Drug Research, Leiden University, Einsteinweg 55, 2333 CC Leiden, The Netherlands; 2 Certara QSP, Canterbury Innovation Centre, Canterbury, UK; 3 Centre for Human Drug Research, Leiden, Netherlands; 4 Leiden University Medical Center, Leiden, The Netherlands

**Keywords:** Boolean model, Sepsis, Toll-like receptor 4, Immune response, Inflammation, Treatment

## Abstract

Sepsis is a life-threatening condition driven by the dysregulation of the host immune response to an infection. The complex and interacting mechanisms underlying sepsis remain not fully understood. By integrating prior knowledge from literature using mathematical modelling techniques, we aimed to obtain a deeper mechanistic insight into sepsis pathogenesis and to evaluate promising novel therapeutic targets, with a focus on Toll-like receptor 4 (TLR4)-mediated pathways. A Boolean network of regulatory relationships was developed for key immune components associated with sepsis pathogenesis after TLR4 activation. Perturbation analyses were conducted to identify therapeutic targets associated with organ dysfunction or antibacterial activity. The developed model consisted of 42 nodes and 183 interactions. Perturbation analyses suggest that over-expression of tumour necrosis factor alpha (TNF-α) or inhibition of soluble receptor sTNF-R, tissue factor, and inflammatory cytokines (IFN-γ, IL-12) may lead to a reduced activation of organ dysfunction related endpoints. Over-expression of complement factor C3b and C5b led to an increase in the bacterial clearance related endpoint. We identified that combinatory blockade of IFN-γ and IL-10 may reduce the risk of organ dysfunction. Finally, we found that combining antibiotic treatment with IL-1β targeted therapy may have the potential to decrease thrombosis. In summary, we demonstrate how existing biological knowledge can be effectively integrated using Boolean network analysis for hypothesis generation of potential treatment strategies and characterization of biomarker responses associated with the early inflammatory response in sepsis.

**Supplementary Information:**

The online version contains supplementary material available at 10.1007/s10928-022-09828-6.

## Introduction

Sepsis is a complex syndrome with high morbidity and mortality associated with multi-organ dysfunction driven by the host inflammatory response to an infection. The initial inflammatory response is mainly activated by pattern recognition receptors, where Toll-like receptor 4 (TLR4) activation is one of the key receptors associated with Gram-negative bacterial infections commonly producing sepsis [1, 2]. Organ dysfunction is a major cause of sepsis-associated mortality and morbidity, although the underlying mechanisms for these effects are only partly understood [3]. Besides treatment with antibiotics, very limited treatment options are currently available for sepsis. Considerable efforts in the past decades towards developing novel therapeutics against sepsis have failed during clinical trials [4, 5]. The complexity of underlying immune system interactions in sepsis in relation to harmful effects on organ systems may be an important reason for these failures, warranting more holistic approaches.

A wealth of knowledge of isolated cellular and biochemical processes and their interactions associated with inflammation and sepsis is available in literature, but the utility of this is hampered by a lack of integration. To this end, the use of mechanistic mathematical modelling may help to integrate this knowledge in order to rationalize the design of treatment strategies, and the discovery of novel biomarkers that may be used to stratify patients and individualize therapies [6, 7]. Indeed, quantitative ordinary differential equation models have been used extensively in systems biology and systems pharmacology for this purpose. However, a requirement for constructing such models is the availability of kinetic parameters, which are lacking for various sepsis and inflammation associated interactions and processes.

Boolean network (BN) models offer an attractive mathematical modelling strategy where inhibitory and stimulatory interactions that are commonly available in literature can be utilized, allowing a much more comprehensive integration of available biological knowledge. BN modelling approaches have been used previously to describe the behaviour of complex systems and to support identification of treatment targets [8, 9]. Briefly, a BN model consists of nodes and edges. Nodes can have an active or inactive state and typically represent biological components such as cells, mediatory molecules or genes [10]. Edges represent the interactions between the different nodes. The BN network is defined according to logic functions that determine the activation state of each node, which will also depend on the activation state of other nodes in the network. Within specific Boolean modelling tools, e.g. *SPIDDOR* [10], interactions between components can also be refined to cause specific activation, inhibition, and modulation of the nodes. Performing simulations with BNs can be used to identify stable states (known as attractors) of the system, which may be considered to correspond to phenotypes [11], thus providing insight into the probability of activation of endpoint nodes with clinical relevance. Comparing the attractors under different perturbations of nodes alone or in combination may be used to identify novel treatment strategies [12].

The aim of this study is to identify cellular or mediator-specific factors which modulate key clinically-relevant endpoints of sepsis, either as explanatory factors of inter-individual variation in treatment outcome, or, as target for potential mono- and combination treatment strategies. To this end, we developed a BN model for the TLR4-mediated host inflammatory response that plays an important role in the systemic inflammatory response in the early phase of sepsis.

## Methods

### Model development

An extensive literature search was performed in order to build the Boolean network model for TLR4-mediated sepsis. We collected experimental in vitro and in vivo data on activation or inhibitory events between key immune cells, intracellular signalling mediatory molecules such as inflammatory cytokines and membrane receptors, and sepsis pathogenesis endpoints including bacterial phagocytosis and thrombosis. The development of the initial version of the model was guided by several comprehensive reviews of the inflammatory response after TLR4 activation and sepsis, from where we systematically searched for each cell and/or mediator for all relevant additional interactions. The BN model was created by translating regulatory interactions between identified cells, receptors and molecules into Boolean functions: interactions of activation or inhibition between nodes were described as flexible combinations of Boolean operators *AND*, *OR* and *NOT* in a mathematical expression. The network was visualized using Cytoscape (v3.8.2) [13].

### Model implementation

The Boolean network analysis was performed in R (v 4.1.2) using the package SPIDDOR (v 1.0) [10], which has multiple essential functionalities for capturing the behaviour of immune responses. One of these functionalities is the introduction of time delays in the network interactions. Such delays are incorporated using threshold arguments (THR) that represent lag times for the initiation of node activation or inhibition. In addition, SPIDDOR allows for modulating the intensity of the activations and inhibitions of the network, by adding a duration for these interactions to occur [10]. In that sense, a regulator node could activate or inhibit the regulated node for only some time steps in the simulation.

To capture the stochasticity associated with biological systems, an asynchronous updating method was implemented for the simulations. This method assumes that only one node can be updated in a single time step and every node is equally likely to be updated [14]. In a BN simulation, each node is updated according to its Boolean function over the time steps, to either remain in, or switch to, one of the two possible states: 0 (inactive) or 1 (activated). The initial state of this BN is the onset of infection (i.e. *Infection* = 1, all other nodes = 0). The state sets of attractors for each simulated scenario, i.e. the percentage of activation (% activation) of each node in 100 repetitions, were used as readout.

### Simulation endpoints

Simulation scenarios were evaluated based on two types of endpoints: the ability of the immune system to fight the infection, through endpoint nodes *Phagocytosis* and *membrane attack complex (MAC)*, and endpoints associated with organ damage, i.e. *Thrombosis* and *angiopoietin-2 (Ang2)*. These aspects could be indirectly represented by nodes in the Boolean network. The extent of activation of these endpoint nodes was used to assess the effect of perturbations of the network.

### Node activation analysis to explore inter-individual variation in clinical endpoints

We studied how variation in immune cell nodes activation can explain differences in activation of selected endpoint nodes to better understand potential causes for variation in clinical outcomes between patients. To this end, we performed a sensitivity analysis by specifying the % activation of each immune cell node in the network from 0 to 100% activation. The sensitivity analysis allowed us to investigate the impact of immune components on clinical endpoint node activation. These analyses were implemented using the polymorphism functionality in SPIDDOR, which modifies the fractional activation patterns of nodes. For instance, when a polymorphism of 50% activity is introduced in a node, this node is only activated 50% of the times in which its regulator nodes are activated [10], therefore, decreasing the normal activity of the node by 50%.

### Perturbation analysis to identify novel mono- and combination treatment targets

Mediatory molecules such as pro-inflammatory cytokines TNF-α and IL-1 are commonly investigated as therapeutic targets in drug development for sepsis [5]. For this analysis, we evaluated the potential of targeting each individual mediatory molecule that was included in the final network. Perturbations were performed via knocking-out or over-expressing a certain mediator node, either at infection onset or at a later stage of the infection until the activation of all nodes in attractors would not change over time steps. We then repeated this analysis where we modulate two nodes at the same time to study the effect of a combination treatment. Here, either we targeted to mediator nodes, or we combined modulation of a mediator with inhibition of the bacterial node to mimic antibiotic treatment. The resulting endpoints activations of attractors were compared to their activations without perturbation. An efficacy cut-off of 20% for the relative activation change between perturbed and non-perturbed scenarios was used to identify promising therapeutic targets.

## Results

### Boolean network development

**Fig. 1:**
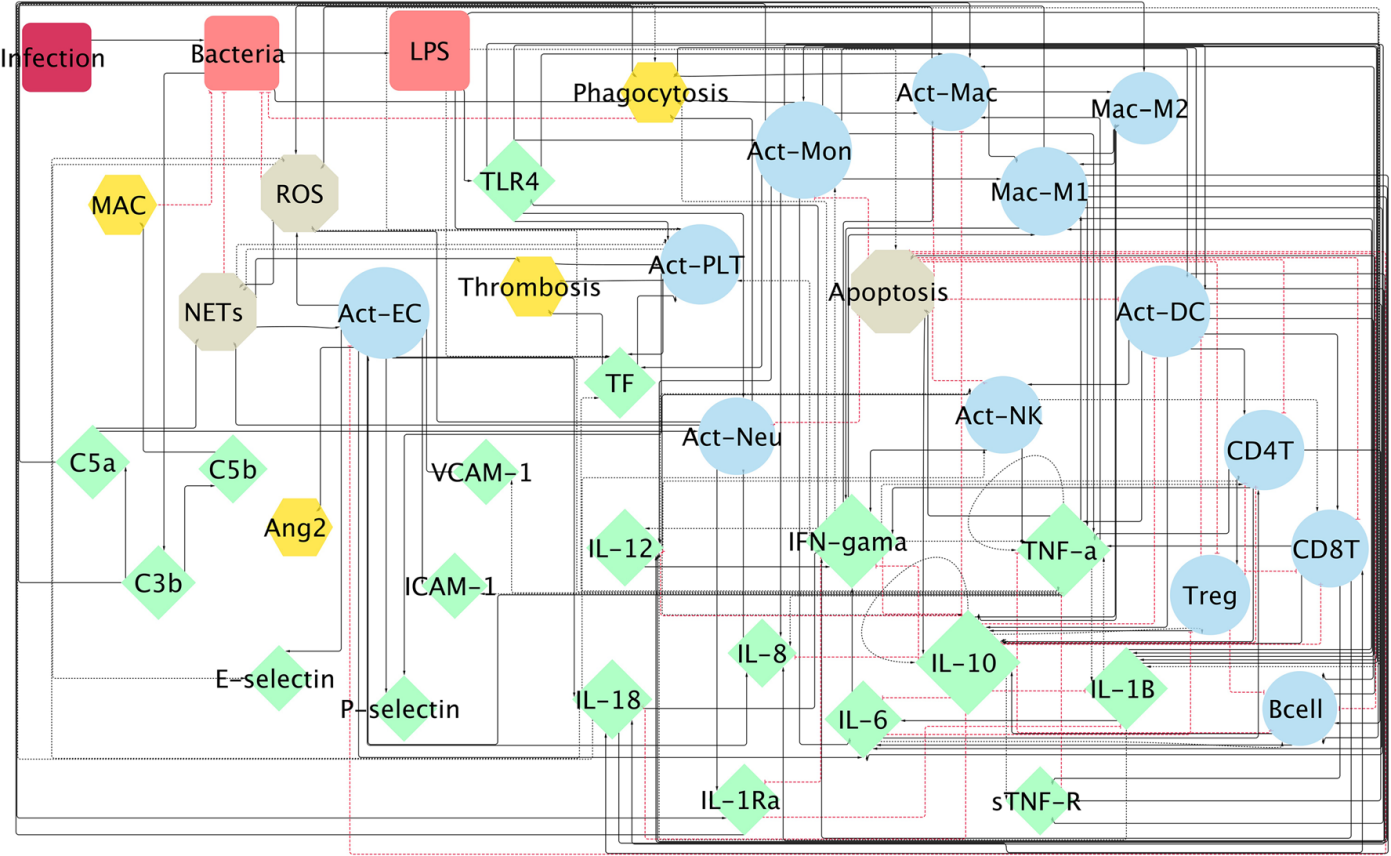
Boolean network model structure representation for the TLR4 activation in early sepsis. Shapes and colors represent different node types, including 3 pathogen related nodes (in red), 13 host cell nodes (in blue), 19 mediator nodes (in green), 4 selected outcome nodes (in yellow) and 3 other nodes (in grey). Size of nodes represents the number of interactions related to a certain node, with the bigger size indicating more interactions. Lines represent the regulations where black solid lines for activation, black dashed lines for positive modulation including auto-secretion and red dashed lines for inhibition (Color figure online)

A Boolean network (Fig. 1) associated with early phase TLR4-mediated sepsis was informed by data extracted from 108 publications (Table S1). The developed BN consisted of 42 nodes and 183 interactions. The underlying Boolean functions are defined further in Table 1 and Table S1. The developed network describes several different mechanisms underlying the disease progression of sepsis, including the regulation of immune cells, endothelial cells, complement and coagulation cascades, which contribute to bacterial clearance but may also lead to activation of harmful effects associated with organ damage. The network used modulations to account for changes in expression, auto-secretion and feedback relationships in a more refined manner (Fig. 1). Threshold parameters (see Methods) were applied to account and differentiate biological time delays for different events, including the clearance of bacteria (*B_CL*), early and late phagocytosis (*Phag_E* and *Phag_L*), cell apoptosis (*Apop*), production of tissue factor (*T_TF*), formation of membrane attack complex (*T_MAC*) and the release of anti-inflammatory cytokines (*Anti_inflam*). Thresholds were set to two time steps to represent the binding and functioning steps, while the threshold relating to early phagocytosis was set to one since it occurs earlier than the phagocytosis caused by other immune cells. The threshold of anti-inflammatory cytokines production, mainly IL-10, was set to three due to an additional required signal transduction for the cytokine synthesis [15].

**Table 1:** Boolean functions of the developed model for TLR4-mediated early sepsis

Nodes	Boolean functions^a^
Infection	Infection = Infection
Bacteria	Bacteria = Infection &! (Bacteria & (THR_MAC[B_CL]^b^ | THR_Phagocytosis[B_CL] | THR_ROS[B_CL] | THR_NETs[B_CL]))
LPS	LPS = Bacteria
TLR4	TLR4 = LPS | (TLR4 & IFN-gamma)
Act-Mon	Act-Mon = (TLR4 | IL-1B | (Act-Mon & IFN-gamma)) &! (Act-Mon & (Apoptosis &! IFN-gamma))
Act-Mac	Act-Mac = (TLR4 | Act-Mon | C5a | (Act-Mac & (TNF-a | IFN-gamma)) | (IFN-gamma & TNF-a) | (Act-Mon & IL-1B)) &! (Act-Mac & (IL-10 | (Apoptosis &! IFN-gamma)))
Mac-M1	Mac-M1 = (Act-Mac & (TNF-a | IFN-gamma)) | (Act-Mon & IL-1B) | (Mac-M2 & (TNF-a | IFN-gamma | IL-1B))
Mac-M2	Mac-M2 = (Act-Mac & (IL-10 | IL-1Ra)) | (Mac-M1 & (IL-10 | IL-1Ra))
Act-DC	Act-DC = (TLR4 | Act-Mon) &! (Act-DC & (Treg | IL-10 | Apoptosis))
Act-Neu	Act-Neu = (TLR4 | C5a | IL-8) &! (Act-Neu & Apoptosis)
Act-NK	Act-NK = (IL-12 | Act-DC | (Act-NK & (IL-12 & IL-18))) &! (Act-NK & Apoptosis)
Act-EC	Act-EC = (TNF-a | NETs) &! (Act-EC & Apoptosis)
Phagocytosis	Phagocytosis = (THR_C3b[Phag_L] & THR_Bacteria[Phag_L]) | THR_Act-Mon[Phag_L] | THR_Act-Mac[Phag_L] | THR_Act-Neu[Phag_E] | THR_Act-DC[Phag_L] | (Phagocytosis & IFN-gamma) | (Phagocytosis & IL-18)
Apoptosis	Apoptosis = THR_TNF-a[Apop] | THR_Bcell[Apop] | (Apoptosis & LPS)
ICAM-1	ICAM-1 = Act-EC | (ICAM-1 &TNF-a)
VCAM-1	VCAM-1 = Act-EC | (VCAM-1 & TNF-a)
E-selectin	E-selectin = Act-EC | (E-selectin & ROS)
P-selectin	P-selectin = Act-EC | Act-PLT
NETs	NETs = Act-Neu | ROS | (Act-Neu & C5a) | (NETs & Act-PLT)
Act-PLT	Act-PLT = LPS | TLR4 | TF | Thrombosis | (Act-PLT & (NETs | IFN-gamma))
TF	TF = THR_Act-Mon[T_TF] | (TF & (THR_Act-EC[T_TF] & (TNF-a | LPS))) | (TF & (THR_Act-Mon[T_TF] & (TNF-a | LPS)))
Thrombosis	Thrombosis = NETs & (TF & Act-PLT)
C3b	C3b = Bacteria
C5a	C5a = C3b
C5b	C5b = C3b
MAC	MAC = THR_C5b[T_MAC]
ROS	ROS = Act-Neu | (Act-Mac | Mac-M1) | Act-EC | (ROS & (TNF-a | IL-18))
Ang2	Ang2 = Act-EC
TNF-a	TNF-a = (Mac-M1 | Act-Mon | Act-NK | Act-DC | CD4T | CD8T | (TNF-a & (IFN-gamma | Act-Mac | ROS )) | (IL-1B & Act-EC)) &! (TNF-a & ((IL-10 &! IFN-gamma) | sTNF-R))
IL-1B	IL-1B = (Act-Mon | Mac-M1 | (IL-1B & (TNF-a | Act-Mon | Act-PLT))) &! (IL-1B & (IL-10 | IL-1Ra))
IFN-gamma	IFN-gamma = (Act-NK | (IFN-gamma & (Act-DC & IL-12)) | (Mac-M1 & (IL-12 & IL-18)) | ((CD4T | CD8T) & (IL-12 | (IL-12 & IL-18))) | (IFN-gamma & (CD4T & IL-6))) &! (IFN-gamma & IL-10)
IL-6	IL-6 = ((Act-Mon & IL-1B) | Mac-M1 | Act-EC | Act-DC | Bcell) &! (IL-6 & IL-10)
IL-8	IL-8 = (Act-Mon | Mac-M1 | Act-EC | (IL-8 & TNF-a)) &! (IL-8 & IL-10)
IL-12	IL-12 = (Act-Mon | Mac-M1 | Act-DC | (IL-12 & (Act-NK | IFN-gamma | IL-1B))) &! (IL-12 & IL-10)
IL-18	IL-18 = (Mac-M1 | Act-DC | Act-EC) &! (IL-18 & IL-10)
IL-10	IL-10 = (THR_Mac-M2[Anti_inflam] | THR_Act-DC[Anti_inflam] | (THR_CD4T[Anti_inflam] | THR_CD8T[Anti_inflam] | THR_Treg[Anti_inflam] | THR_Bcell[Anti_inflam]) | (IL-10 &(Act-DC | IL-12)) | THR_Apoptosis[Anti_inflam]) &! (IL-10 & IFN-gamma)
sTNF-R	sTNF-R = THR_Act-Mon[Anti_inflam] | THR_CD4T[Anti_inflam] | THR_CD8T[Anti_inflam] | (sTNF-R & IL-10)
IL-1Ra	IL-1Ra = (THR_Act-Neu[Anti_inflam] | THR_Act-Mon[Anti_inflam]) &! (IL-1Ra & IFN-gamma)
CD4T	CD4T = (Act-DC | IL-6 | (CD4T & (IL-12 | IFN-gamma))) &! (CD4T & (IL-10 | Treg | Apoptosis))
CD8T	CD8T = (Act-DC | IL-18 | (CD8T & Act-NK)) &! (CD8T & (Treg | IL-10 | Apoptosis))
Treg	Treg = (CD4T | (Treg & IL-10)) &! (Treg & (IL-6 | Apoptosis))
Bcell	Bcell = (TLR4 | (Act-DC & LPS) | (Bcell & IL-6)) &! (Bcell & (Treg | IL-10 | Apoptosis))

^a^Boolean functions were mathematical expressions with different nodes and flexible combinations of logic operators AND (&), OR (|) and NOT (!), where “&” and “|” mainly represented for different activation mode and “!” for inhibition. For example, the Boolean function for node bacteria, “Bacteria = Infection &! (Bacteria & (THR_MAC[B_CL] | THR_Phagocytosis[B_CL] | THR_ROS[B_CL] | THR_NETs[B_CL]))”, means bacteria appears upon infection while either membrane attack complex or host cell phagocytosis or reactive oxygen species or neutrophil extracellular traps works to clear bacteria with certain time delays. Definitions of all nodes and related regulatory interactions were shown in supplemental Table S1

^b^Threshold arguments were shown in [ ] referring to time delay, where for bacterial clearance (B_CL), late phagocytosis (Phag_L), apoptosis (Apop), membrane attack complex (T_MAC) and tissue factor (T_TF) the thresholds were set as 2, for early phagocytosis (Phag_E) and anti-inflammatory markers (Anti_inflam) thresholds were set as 1 and 3, respectively

### Node activation analysis to explore inter-individual variation in clinical endpoints

**Fig. 2:**
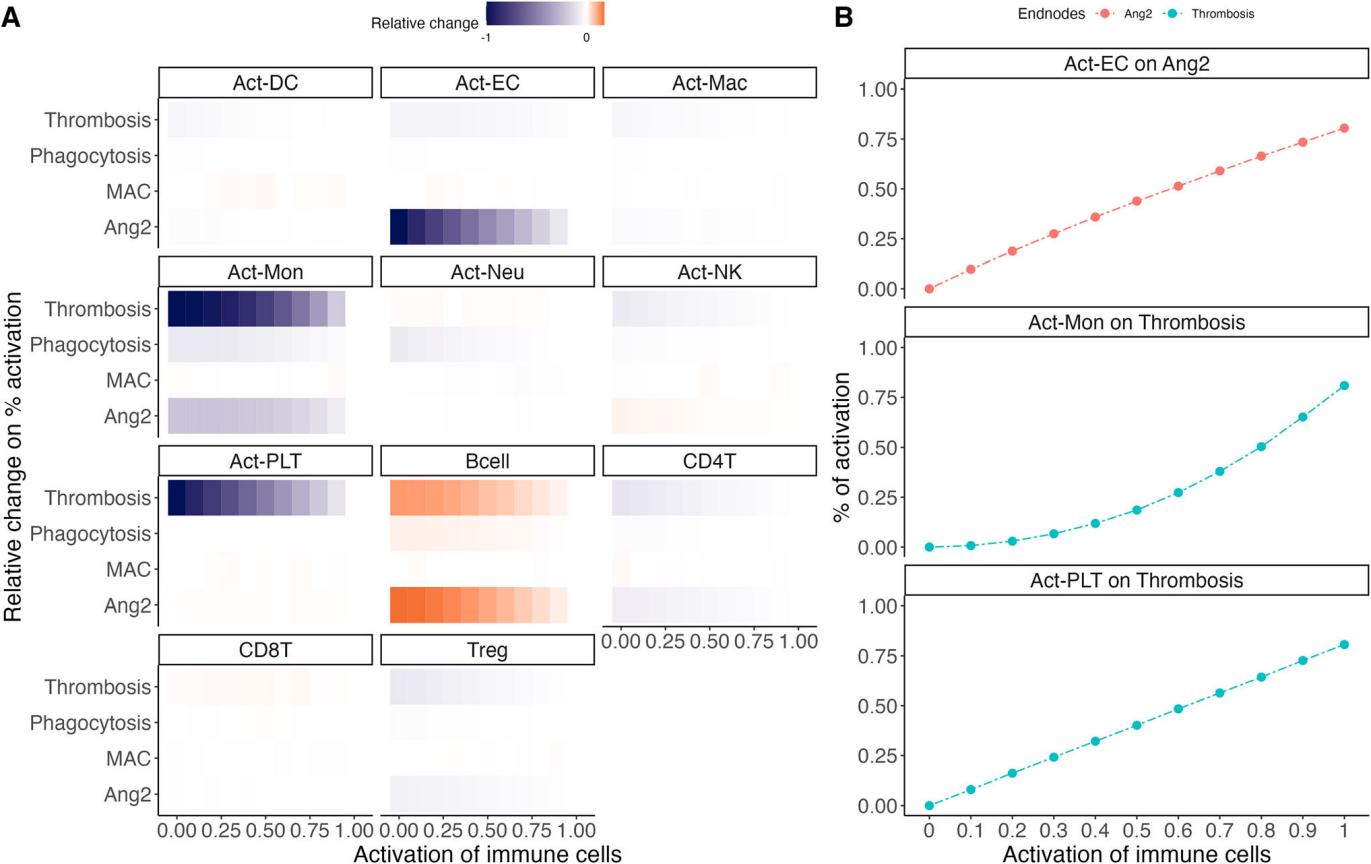
Sensitivity analysis of the effect of immune cells activation on four selected endpoints. The heatmap (**A**) showed the effect of decreased activation of different immune cells, ranging from 0 (deactivated) to 100% (normal activation), on four endpoints compared with their normal activation pattern (100% activation), colors of the heatmap represented the negative, neutral and positive relative changes of endpoints % activation on attractors with blue, white and orange, respectively; The scatter plot (**B**) with lines showed three identified effects of immune cells on selected endpoints: activation variation of activated endothelial cells (*Act-EC*) on angiopoietin-2 (*Ang2*), and activation variations of activated monocytes (*Act-Mon*) and activated platelets (*Act-PLT*) on thrombosis (*Thrombosis*). Effect of B cells (*Bcell*) activation on *Thrombosis* and *Ang-2* were not identified due to the small relative changes of % activation of endpoints on attractors (Color figure online)

We performed a sensitivity analysis to evaluated the impact of node activation alterations on innate and adaptive immune cell nodes as well as activated endothelial cells, by performing simulations where we decreased the activation of these nodes a 10% in each simulation and then compared the effect caused on the endpoints with the state of these endpoints on attractors with no alteration (100% activation). As a result, we identified three cell nodes whose activation situation had considerable effect on the selected endpoints: (1) activated endothelial cells (*Act-EC*) on angiopoietin-2 (*Ang2*), (2) activated monocytes (*Act-Mon*) on thrombosis (*Thrombosis*), and (3) activated platelets (*Act-PLT*) on thrombosis (*Thrombosis*) (Fig. 2**)**.

The activation level of endothelial cells was positively correlated with angiopoietin-2 activity, with higher activation of *Act-EC* as initial state leading to a higher activation of *Ang2* on attractors. This finding is in line with previous studies, where activated endothelial cells have been shown to release more angiopoietin-2 into circulation during inflammation compared to non-inflammatory condition [16]. The risk of thrombosis, i.e., activation of the *Thrombosis* node in our network, was correlated with increasing monocyte and platelet activation. These two cell types play key roles in thrombosis as monocytes are the direct production source of tissue factor (TF) [17], while TF and platelet activation form the very foundation of thrombotic events. The increased activation of platelets could partly explain the increased risk of thrombosis and thromboembolism seen in the elderly patients [18].

### Perturbation analysis to identify novel mono- and combination treatment targets

We compared the relative change (Eq. 1) in % activation of endpoints between scenarios with different perturbation initiation times. In this analysis, we found that the relative changes were similar over perturbation initiation time in both singular and combination perturbation analysis. The result may indicate that variation in timing of the perturbation does not lead to relevant differences on the % activation on attractors of our selected endpoints (Fig. S1A–B).

1 $${\text{Relative change}} = ~\frac{{\%\,{\text{activation under perturbation}} - \%\,{\text{activation without perturbation}}}}{{\%\, {\text{activation without perturbation}}}}$$

**Fig. 3:**
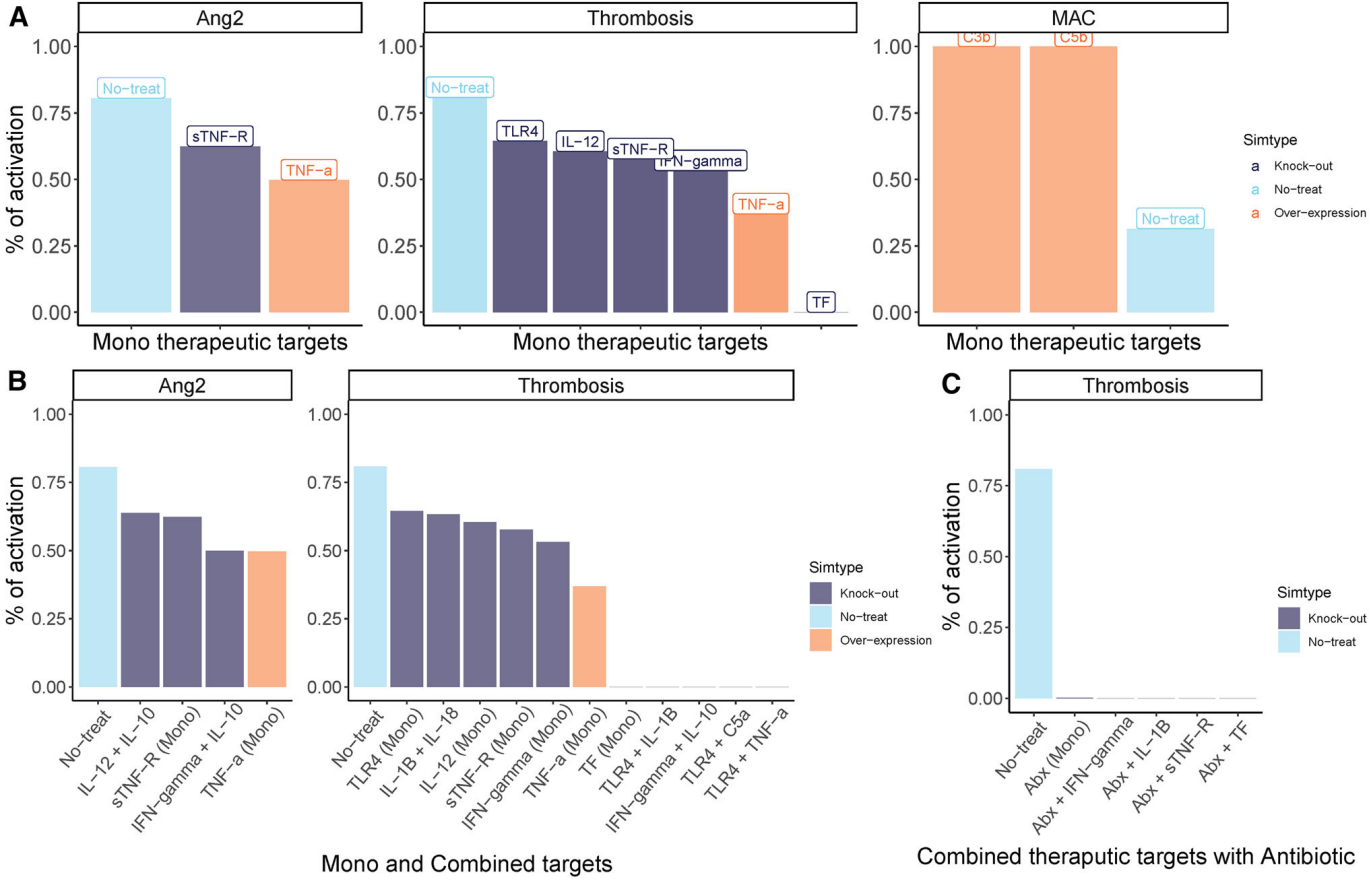
Therapeutic targets identified through perturbation analyses. The bar plots **A** represented the effect of selected mono-therapeutic targets on endpoints; **B** represented the effect of both mono and combine-therapeutic targets on their corresponding endpoints; **C** represented the effect of antibiotic and/or combined therapeutic target with antibiotic on endpoint *Thrombosis*. Colors of the bar plots represented the no perturbation, knocking out and over expression with blue, dark blue and orange, respectively (Color figure online)

We identified a set of potential mono-therapeutic targets that were associated with a decreased activation of *Ang2* and *Thrombosis* and/or to increase *MAC* (Fig. 3A). Two targets (*sTNF-R* and *TNF-a)* were identified for *Ang2*, six targets (*IL-12*, *sTNF-R*, *IFN-gama*, *TNF-a* and *TF*) were selected for *Thrombosis*, and two targets (*C3b* and *C5b*) were selected for *MAC*. No single perturbation displayed an impact on *Phagocytosis* based on our evaluation criteria. Furthermore, we found that either over-expressing tumour necrosis factor alpha (TNF-α) or blocking soluble TNF receptor (sTNF-R) could lead to a reduction of both of the organ dysfunction endpoints (*Ang2* and *Thrombosis*). Blocking TLR4, TF or inflammatory cytokines interferon (IFN)-γ or interleukin (IL)-12 could reduce the risk of thrombosis but showed no beneficial effect on reducing angiopoietin-2. For the bacterial clearance related endpoint *MAC*, the over-expression of complement component C3b and C5b showed to increase its average long-term activation. This is in line with the well-established role of C5b as an essential composition of membrane attack complex (MAC) and that the cleavage of C5 to C5b requires C3b [19]. Although the interaction between the complement system and MAC is not an unexpected finding, it adds towards building confidence in the model predictions.

We identified a total of six multi-target treatment strategies that showed potential benefit (Fig. 3B) in which one combination, by blocking IFN-γ and IL-10 together, could reduce both the risk of thrombosis and vessel leakage which is represented by activation of node *Ang2* based on our network. Another combination shown to decrease the activation of *Ang2* was blocking cytokines IL-10 and IL-12 together. Three of all the other four combinations to decrease the activation of *Thrombosis* included targeting TLR4, while the last one relied on the simultaneous blocking of IL-1β and IL-18. For therapy directed towards improving bacteria clearance by increasing *MAC* or *Phagocytosis*, no effective combinations were identified.

When combining an immune targeting therapy with antibiotic treatment, where the antibiotic has a rapid and direct effect on bacterial clearance, the timing of initiation of treatment is of importance. The effect of clearing bacteria based on our selected endpoints differed over time and showed to be most beneficial during the early stage of infection (i.e. before 4 time steps, Fig. S2). This finding adds to the evidence of rapid initiation of antibiotic therapy improves outcomes in septic patients [20]. Although the use of antibiotics as mono-therapy showed a reduction of *Thrombosis* activation by more than 20%, our perturbation analysis suggests that there are still potential beneficial options of combining antibiotics with a *Thrombosis* focused therapy.

Overall, we identified four therapeutic targets that could be beneficial to target in combination with antibiotic therapy to decrease the activation of *Thrombosis* (Fig. 3C**)**, in which three of them were already identified in mono-therapy evaluation, i.e. IFN-γ, sTNF-R or TF, but blocking them could almost deactivate *Thrombosis* when combined with antibiotics. Another identified target, pro-inflammatory cytokine IL-1β, was not identified in mono-therapy but appeared in combined mediators specific therapies. For node *Ang2*, no combination showed a benefit to decrease its activation. Predictably, no increased immune regulated antibacterial effect could be identified due to the rapid bacterial eradication mediated by the antibiotics.

## Discussion

A novel Boolean network model was developed, which leveraged prior knowledge of immune response-related processes for the TLR4-mediated host response associated with early phase sepsis. The developed network incorporated key immune cells and mediatory molecules, as well as key clinical endpoint nodes to assess inter-individual variability and treatment interventions. By using a simulation approach, we identified several potential targets showing promise of improving bacterial clearance and/or reducing the possibility of organ dysfunction. The identified mediators might constitute potential therapeutic targets for treatment of sepsis and could be considered in further clinical studies.

The long-term behaviour, i.e. attractors, of this developed network showed to be stable according to the overall single perturbation analysis, where either knocking-out or over-expression of most nodes did not trigger considerable changes on the activation of the rest nodes on attractor (Fig. S3). This stabilization could be a result of the complex interactions within the network, which might explain in part the failures of many clinical trials investing treatments against sepsis. Recently, selective or non-selective targeting of endogenous mediator molecules have been investigated as strategies to modify the systemic inflammatory response, such as blocking TNF-α and IL-1β [4, 5]. However, none of these agents showed significant improvement on septic survival rate. These results are comparable to our single node perturbations in which knocking-out TNF-α mainly lead to decreasing cell apoptosis while knocking-out IL-1β showed no big influence on other nodes.

We utilized a Boolean network as a tool to screen promising treatment targets for sepsis based on endpoints related to bacteria clearance (*Phagocytosis* and *MAC*) and vessel leakage and multi-organs dysfunction (*Ang2* and *Thrombosis*). When evaluating mono-target therapies, we found over-expressing TNF-α, instead of blocking it, was associated with a decreased activation of *Ang2* and *Thrombosis*, which can be related to decreased organ dysfunctions. This finding is inconsistent with previous clinical studies where TNF-α was blocked but have not shown a significantly improved survival rate in sepsis patients [5]. Additionally, treatments blocking either TF or IFN-γ were identified to reduce *Thrombosis* in our analysis. These targets have also been studied in clinical trials, but so far no clinical effect has been identified [4]. One reason for these inconsistent results might be the differences in selected endpoints. Clinical trials for sepsis mainly use mortality as the primary endpoint, while we used four surrogate endpoints.

Our simulations suggest a decrease in activation of *Ang2* after over-expressing TNF-α. In contrast, a previous in vitro study suggested TNF-α can induce both angiopoietin-2 mRNA expression and protein levels in human umbilical vein endothelial cells [21] at 2 h after TNF-α exposure. Importantly, the positive interaction between angiopoietin-2 and TNF-α is in fact included in our model, with activated endothelial cells as intermediate node (Table 1). However, unlike the in vitro experiment involving a single cell type, our Boolean model also incorporates other relevant interaction events derived from other additional experiments, thereby illustrating the value of deriving expected outcomes which are the results of multiple cellular interaction events.

The effect of TNF-α on thrombosis remains inconclusive. Previous studies suggested either an antithrombotic activity through the stimulation of nitric oxide [22] or a prothrombotic effect via acting on TNF-α receptor subtype 2 [23]. Recently, a in vivo study in mice showed a positive regulation of TNF-α/TNF receptor p55 singling axis in the resolution of venous thrombus [24]. In our simulations, long term over-expression of TNF-α was likely to decrease the activation of node *Thrombosis*, which might be a result of its beneficial role in thrombus resolution as indicated in the animal study. Worth noting are the inevitable inter-species differences when using animal models to mimic pathophysiological features in humans [25].

For multi-target treatment strategies, the combination of blocking IFN-γ and IL-10 was identified as a potential treatment to decrease the risk of organ dysfunction, via reducing activation of both *Ang2* and *Thrombosis*. Cytokine IFN-γ functions as a positive modulator of activated platelets [26], which plays a crucial role in the process of thrombosis. Although IL-10 shows an inhibitory effect on the production of most pro-inflammatory cytokines, increased IL-10 blood levels has been associated with the development of organ failure in septic shock [27]. Nevertheless, since IFN-γ and IL-10 are negative modulators of each other, few studies have addressed the co-operative action of these combination, while Yoshiki et al. found simultaneous treatment with IL-10 and IFN-γ can significantly suppress the function of murine bone marrow-derived dendritic cells [28]. Due to the complexity of regulatory interaction between cytokines, the blockage of IFN-γ and IL-10 together could potentially reduce the risk of organ dysfunction.

When treating with antibiotics in the very early phase of infection, all nodes in this network remained inactive or returned to baseline immediately (Fig. S2). This behaviour is in line with the clinical recommendation of administering antibiotics as early as possible for adults with possible septic shock or a high likelihood for sepsis [2]. A delayed start of antibiotic therapy, simulated by removing bacteria after 4 time steps, showed to be ineffective in inhibiting the initiation of the immune cascade reaction, which can be seen from the unchanged activation on attractors (Fig. S4). This phenomenon may explain the failures of clinical trial focusing on anti-endotoxin agents [5], where neither human antiserum to endotoxin nor monoclonal IgM antibodies that inactivates endotoxin could significantly improve survival in sepsis.

Local thrombosis contributes to the initial defence against bacterial invasion in mammals [29]. We find that combination therapies with delayed initiation of antibiotic therapy, such as antibiotic treatment combined with IL-1β blockade, may show beneficial effects, decreasing *Thrombosis* node activation. These results are in line with a previous study where an increase in IL-1β mRNA expression in patients who suffered thrombotic episodes compared with healthy age-matched controls [30] was observed. Another clinical study showed the anti-inflammatory therapy targeting IL-1β pathway led to a significantly lower rate of recurrent cardiovascular events than placebo [31]. These data indicate that IL-1β might be a relevant therapeutic target, although treatment of inhibiting IL-1β alone did not show sufficient decrease of *Thrombosis* activation in our analysis.

Interleukins have been of recent interest as potential treatment in sepsis due to their contribution to thrombosis and their potential therapeutic effect in animal models [32], including pro-inflammatory IL-6 [33] and anti-inflammatory IL-10 [34]. However, a population-based study suggested that an altered inflammatory profile of these interleukins is more likely to be associated with a result rather than an increased risk of venous thrombosis [35]. IL-12 was another identified target in our simulations. However, a previous study concluded that IL-12 can activate both coagulation and fibrinolysis in patients with renal cell carcinoma [36]. The potential of these inflammatory targets thus still need to be evaluated in well-controlled clinical studies.

Antibiotic treatment was mimicked by setting the node *Bacteria* to 0% activation in our simulation. The dynamic pattern over time steps of other nodes varies after deactivating *Bacteria* (Fig. S2), in which the simulated activation of complement factors, i.e., *C3*, *C5a* and *C5b*, as well the complex *MAC* returned to baseline immediately. This consistency indicates the potential of complement factors as biomarkers for monitoring antibiotic treatment efficacy in early sepsis. Indeed, a recent prospective study evaluated complement levels in bacteremia patients, and hypothesized the measurement of C3, C4 and C9 levels may help stratify Gram-negative bacteremia patients at increased risk for mortality [37]. Activation of complement system is a key event in the pathogenesis of sepsis [38], adapting crucial complement factors as biomarkers might be of prognostic value, when their sensitivity and specificity were carefully evaluated.

Although the use of a Boolean network approach can support developing understanding the behaviour of complex systems, especially in the lack of quantitative data, the approach is associated with inherent limitations. The time steps in a Boolean network are not related to real time. Thus, simulation results cannot be directly linked to time-concentration data, such as specific biomarker peak times, which further complicates model validation using clinical data. The attractors of mono perturbations on our BN were compared with previous experimental results under certain intervention, revealing some similarities between our simulations and in vivo animal studies. However, human studies with comparable endpoints are still required to validate both of the identified mono and multi therapeutic targets.

The development of the Boolean network model in this study was guided by including key biological processes previously identified as key consensus mechanisms associated with TLR4-activation and early sepsis. We systematically searched the literature to identify interaction partners between involved cell types, receptors and their ligands to populate a complete network. Nonetheless, the developed Boolean network model may need further revision and additions depending on new findings and specific objectives for applying this model. With respect to (clinical) endpoint nodes we have selected biological events which may closely relate to key clinical events in the disease pathology of sepsis. Yet, it is important to recognize this model does not directly predicts clinical outcomes, which also complicates the comparison of our results to existing clinical trials. These two shortcomings could be overcome by gradually extending this network with a higher number and clinically related nodes.

In conclusion, the developed Boolean network model for TLR4-mediated host immune response in early phase of sepsis exemplifies the value of using Boolean networks to increase the knowledge of complex biological systems, and constitutes a relevant strategy to deepen our understanding of systemic inflammatory diseases, analyse the influences of immune cells diversity among patient groups, and identify potential therapeutic targets for sepsis.

## Supplementary Information

Below is the link to the electronic supplementary material.

Supplementary material 1 (DOCX 183.5 kb)

Supplementary material 2 (TIF 2038.8 kb)
**Fig. S1** Relative changes of four selected endpoints activation under mono and combined perturbations on mediatory molecules. Upper heatmap (A) showed the effect of knocking-out or over-expressing of identified mono therapeutic targets on four endpoints over different perturbation initiation time steps; below heatmaps (B) showed the example of effect of knocking-out or over-expressing combined therapeutic targets on four endpoints when initiating perturbations at time step 20. Colors of the heatmap represented the negative, neutral and positive relative changes of endpoints activation with blue, white and orange, respectively

Supplementary material 3 (TIFF 2204.7 kb)
**Fig. S2** Average activation profiles for each node under antibiotic treatment (i.e. knocking out node *Bacteria*) at different time step with 100 repetitions. When removing bacteria at an early phase (before time step 4), most nodes were not activated or returned back to baseline immediately; when removing bacteria at a later phase, it showed varying decline patterns for different nodes. Colors of the lines represented different perturbation initiation time steps

Supplementary material 4 (TIF 928.5 kb)
**Fig. S3** Overview of single node perturbation analysis of the network. The heatmaps indicated the effect of entire knock-out (A) or over-expression (B) of each node (columns) in every network node (rows). Colors of the heatmap represented the Perturbation Index (PI) with the negative (PI < 0.8), neutral (0.8 < PI < 1.25) and positive (PI > 1.25) changes being blue, white and orange, respectively

Supplementary material 5 (TIFF 756.0 kb)
**Fig. S4 **Activation of each node on attractors under antibiotic treatment (i.e. knocking out node *Bacteria*) at different time steps. The activations on attractors stayed unchanged when removing bacteria at a later phase (i.e. after time step 4)
