# Supplementary material for: A system pharmacology Boolean network model for the TLR4-mediated inflammatory response in early sepsis
Source: J Pharmacokinet Pharmacodyn. 2022 Oct 19;49(6):645–55. doi: 10.1007/s10928-022-09828-6 (PMC9649476; doi:10.1007/s10928-022-09828-6)
Supplement: Supplementary file 1 — Supplementary material 1 (DOCX 183.5 kb) [file 10928_2022_9828_MOESM1_ESM.docx]

# A system pharmacology Boolean network model for the TLR4-mediated inflammatory response in early sepsis

F. Liu^1^, L.B.S. Aulin^1^, S. Kossen^1^, J. Cathalina^1^, M. Bremmer^1^, A. Foks^1^, P.H. van der Graaf^1,2^, M. Moerland^3,4^, J.G.C. van Hasselt^1*^

^1^Leiden Academic Centre for Drug Research, Leiden University, Leiden, The Netherlands.
^2^Certara QSP, Canterbury Innovation Centre, Canterbury, United Kingdom.

^3^Centre for Human Drug Research, Leiden, Netherlands

^4^Leiden University Medical Center, Leiden, The Netherlands

Keywords: Boolean model, sepsis, toll-like receptor 4, immune response, inflammation, treatment

Abstract: 222 words

Manuscript: 3298 words

References: 23 + 108 (supplemental)

Figures: 3 + 4 (supplemental)

Tables: 1 + 1 (supplemental)

*Corresponding author:

Coen van Hasselt, Leiden University, Einsteinweg 55, 2333 CC, Leiden, The Netherlands.

Tel.: +31 0648216727, Email: coen.vanhasselt@lacdr.leidenuniv.nl

**Table S1** **Definitions of all nodes and related regulatory interactions in the final Boolean network**

| Node | Explanation | Dependency | Interaction | Refs |
| --- | --- | --- | --- | --- |
| Infection | Bacterial infection | Infection | Initial node, always ON after sepsis onset |  |
| Bacteria | Gram-negative bacteria | Infection | Sepsis is mostly caused by Gram-negative bacteria infection. Infection ON means there are bacteria |  |
|  |  | MAC | MAC can form cytotoxic pores on the surface of microbes and directly kill Gram-negative bacteria | [1] |
|  |  | Phagocytosis | Phagocytosis is a cellular process for ingesting and eliminating microorganisms, foreign substances, and apoptotic cells | [2] |
|  |  | ROS | The first discovered function of ROS in neutrophils was their microbicidal activity | [3] |
|  |  | NETs | NET killing can be as effective as phagocytosis | [4] |
| LPS | Lipopolysaccharides | Bacteria | The LPS is a central component of the outer membrane in Gram-negative bacteria and plays a key role in pathogenesis | [5] |
| TLR4 | Toll-like receptor 4 | LPS | Several Pathogen-associated molecular patterns can stimulate TLR4 including LPS | [6] |
|  |  | IFN-gamma | In human monocytes and macrophages, IFN-γ augmented mRNA and surface expression of TLR4 | [7] |
| Act-Mon | Activated Monocyte | TLR4 | The number of monocytic TLR-4 expression were markedly increased in sepsis | [8] |
|  |  | IL-1B | Primary blood monocytes are activated by mature IL-1β | [9] |
|  |  | IFN-gamma | IFNγ-primed monocytes showed increased responsiveness to LPS | [7] |
|  |  | Apoptosis | Apoptosis of stimulated monocytes is inhibited by IFN-γ | [10] |
| Act-Mac | Activated Macrophage | TLR4 | TLRs are highly expressed on macrophages | [11] |
|  |  | Act-Mon | During inflammation, circulating monocytes migrate into tissues, differentiate into macrophage | [12] |
|  |  | C5a | Complement anaphylatoxin C5a trigger macrophage activation | [13] |
|  |  | TNF-a | TNF-α amplifies inflammatory cascades by activating macrophages | [14] |
|  |  | IFN-gamma | IFN-γ promotes innate immune responses by activating macrophages | [15] |
|  |  | IL-1B | IL-1β induces the rapid differentiation of monocytes into CD209+ macrophages | [16] |
|  |  | IL-10 | IL-10 inhibits cytokine production by activated macrophages | [17] |
|  |  | Apoptosis | Apoptosis of stimulated macrophages is inhibited by IFN-γ | [10] |
| Mac-M1 | M1-like Macrophage | Act-Mac | M1-like macrophage is a subset of macrophages | [18] |
|  |  | TNF-a | TNF-α promotes the activation and differentiation of macrophages | [19] |
|  |  | IL-1B | IL-1β promotes the differentiation of monocytes into M1-like macrophages | [16] |
|  |  | IFN-gamma | M1 macrophages are typically induced by Th1 cytokines, such as IFN-γ and TNF-α | [19] |
|  |  | Mac-M2 | “Re-polarization” of differentiated macrophages includes M2 macrophages to M1 signals, or vice versa | [19] |
| Mac-M2 | M2-like Macrophage | Act-Mac | M2-like macrophage is a subset of macrophages | [18] |
|  |  | IL-10 | IL-10 promotes M2 polarization | [20] |
|  |  | IL-1Ra | IL1Ra control the polarization of macrophages toward a M2 phenotype | [21] |
|  |  | Mac-M1 | “Re-polarization” of differentiated macrophages includes M2 macrophages to M1 signals, or vice versa | [19] |
| Act-DC | Activated Dendritic Cell | TLR4 | TLR4 is essential for dendritic cell activation | [22] |
|  |  | Act-Mon | Monocytes can further differentiate into a range of tissue dendritic cells | [12] |
|  |  | Treg | Regulatory T-cells mediate their suppressive action by acting on dendritic cells | [23] |
|  |  | IL-10 | IL-10 is a critical cytokine that blocks the maturation of dendritic cells (DCs) | [24] |
|  |  | Apoptosis | Apoptotic cell death plays an important role in inflammatory processes | [10] |
| Act-Neu | Activated Neutrophil | TLR4 | Activation of TLR4 caused changes in adhesion molecule expression on neutrophils | [25] |
|  |  | C5a | C5a generated during systemic inflammatory processes activates neutrophils | [26] |
|  |  | IL-8 | IL-8 was originally discovered as chemokines activating neutrophil granulocytes | [27] |
|  |  | Apoptosis | Apoptotic cell death plays an important role in inflammatory processes | [10] |
| Act-NK | Activated Natural Killer Cell | IL-12 | IL-12 directly generated high lymphokine-activated killer cell activity in CD56+ NK cells | [28] |
|  |  | IL-18 | The combination of IL-18 and IL-12 induced extremely high amounts of IFN-γprotein secreted by NK cells | [29] |
|  |  | Act-DC | Both immature and mature DCs activate resting human natural killer cells | [30] |
|  |  | Apoptosis | Apoptotic cell death plays an important role in inflammatory processes | [10] |
| Act-EC | Activated Endothelial Cell | NETs | NETs promote the activation of Endothelial Cells | [31] |
|  |  | TNF-a | Type II activation of endothelial cells mediated by pro-inflammatory cytokines such as tumor-necrosis factor | [32] |
|  |  | Apoptosis | Apoptotic cell death plays an important role in inflammatory processes | [10] |
| Phagocytosis | Phagocytosis | Act-Mon | Professional phagocytes accomplish phagocytosis with high efficiency, including Macrophages, neutrophils, monocytes, and dendritic cells | [2] |
|  |  | Act-Mac | Professional phagocytes accomplish phagocytosis with high efficiency, including Macrophages, neutrophils, monocytes, and dendritic cells | [2] |
|  |  | Act-Neu | Neutrophils are typically the first non-resident immune cells that arrive at a site of inflammation and its phagocytosis is an early event | [33] |
|  |  | Act-DC | Professional phagocytes accomplish phagocytosis with high efficiency, including Macrophages, neutrophils, monocytes, and dendritic cells | [2] |
|  |  | Bacteria | Complement labels bacteria with C3-derived products (C3b and C3bi) stimulate engulfment of bacteria by phagocytes. | [34] |
|  |  | C3b | Complement labels bacteria with C3-derived products (C3b and C3bi) stimulate engulfment of bacteria by phagocytes. | [34] |
|  |  | IFN-gamma | Complement receptors of mononuclear phagocytes are up-regulated by IFN-γ to promote receptor-mediated phagocytosis of opsonized extracellular pathogens. | [35] |
|  |  | IL-18 | IL-18 restored the burn-related decrease in activity of neutrophils and enhanced phagocytosis | [36] |
| Apoptosis | Apoptosis | TNF-a | TNF-induced apoptosis primarily through the activation of type I receptors | [37] |
|  |  | Bcell | Mechanisms of BCR-mediated apoptosis have been widely studied in B cells | [38] |
|  |  | LPS | LPS induces cell death as measured by caspase-3 activation and DNA fragmentation | [39] |
| ICAM-1 | Intercellular Adhesion Molecule 1 | Act-EC | Cytokine-activated venular endothelial cells increase their expression of ICAM1 | [32] |
|  |  | TNF-a | TNF-α enhances the expression of adhesion molecules | [14] |
| VCAM-1 | Vascular cell adhesion molecule 1 | Act-EC | Cytokine-activated venular endothelial cells increase their expression of VCAM1 | [32] |
|  |  | TNF-a | TNF-α enhances the expression of adhesion molecules | [14] |
| E-selectin | E-selectin | Act-EC | E-selectin expressed on activated endothelial cells | [40] |
|  |  | ROS | Generation of ROS in endothelial cells is a critical signal mediating E-selectin expression. | [41] |
| P-selectin | P-selectin | Act-EC | P-selectin expressed on activated endothelial cells | [40] |
|  |  | Act-PLT | P-selectin expressed on activated platelets | [40] |
| NETs | Neutrophil extracellular traps | ROS | ROS can activate granular proteases and induce the formation of NETs | [42] |
|  |  | Act-Neu | Activated neutrophils release nuclear DNA and form a network structure NETs | [31] |
|  |  | C5a | C5a pre-stimulated neutrophils enhanced their ability to generate NETs | [31] |
|  |  | Act-PLT | The ability of platelets to promote NET formation was also observed in human cells | [43] |
| Act-PLT | Activated platelet | LPS | LPS can initiate the non-classical activation of platelets | [31] |
|  |  | TLR4 | TLR4 was found to activate platelet through different pathways | [44] |
|  |  | TF | Platelet activation is stimulated by local prothrombotic factors such as tissue factor | [45] |
|  |  | Thrombosis | Thrombin activates platelets through protease-activated receptors | [45] |
|  |  | NETs | NETs further promote platelet activation | [31] |
|  |  | IFN-gamma | IFN-γ act on platelets to promote the degranulation of dense granules | [46] |
| TF | Tissue factor | Act-Mon | monocytes expressed TF mRNA in human model of endotoxemia | [47] |
|  |  | Act-EC | In ECs TNF-α and LPS are shown to up-regulate TF expression in vitro | [48] |
|  |  | LPS | LPS and TNF-a can also up-regulate TF expression from monocyte | [48] |
|  |  | TNF-a | LPS and TNF-a can also up-regulate TF expression from monocyte | [48] |
| Thrombosis | Thrombosis | NETs | The interaction of NETs with platelets mediates the formation of immune thrombosis | [31] |
|  |  | Act-PLT | The interaction of NETs with platelets mediates the formation of immune thrombosis | [31] |
|  |  | TF | In pathological conditions, elevated levels of tissue factor and deposition of platelets may conspire to trigger formation of a thrombotic clot | [49] |
| C3b | Component 3b | Bacteria | Bacteria have evolved several modulators of initial steps in complement activation to generate C3 convertases, the enzymes that cleave C3 to C3b | [50] |
| C5a | Component 5a | C3b | C3b binds to the C3 convertase, forming the C5 convertase enzyme cleaving C5 into C5a and C5b | [51] |
| C5b | Component 5b | C3b | C3b binds to the C3 convertase, forming the C5 convertase enzyme cleaving C5 into C5a and C5b | [51] |
| MAC | Membrane attack complex | C5b | Stepwise MAC assembly pathway from soluble complement factors requires the large fragment C5b | [1] |
| ROS | Reactive oxygen species | Act-Neu | Neutrophils will release large amounts of ROS at the site of infection following the activation of surface receptors | [42] |
|  |  | Act-Mac | ROS formation in macrophages from Cytosolic or Mitochondrial sources | [52] |
|  |  | Mac-M1 | M1 macrophages participate in subsequent generation of ROS | [19] |
|  |  | Act-EC | Increased generation of ROS are among the major molecular changes associated with endothelial dysfunction | [53] |
|  |  | TNF-a | Mitochondrial ROS production increased after TNF-α stimulation | [54] |
|  |  | IL-18 | IL-18 strongly enhances the induction of ROS in phagocytes. | [36] |
| Ang2 | Angiopoietin-2 | Act-EC | Expression profiling studies have identified endothelial cells as the primary source of Ang-2 and a dramatic transcriptional regulation of Ang-2 production on endothelial cell activation | [55] |
| TNF-a | Tumor necrosis factor alpha | Act-Mon | TNF-α produced predominantly by activated monocytes/macrophages | [56] |
|  |  | Mac-M1 | These M1 type macrophages produce and secrete higher levels of TNF-α | [19] |
|  |  | Act-DC | DC maturation result in secretion of the inflammatory cytokines TNF-α. | [57] |
|  |  | Act-NK | NK cells also secrete several cytokines such as IFN-γ and TNF-α | [58] |
|  |  | CD4T | TNF is a product of effector CD4 and CD8+ T cells or innate cells | [59] |
|  |  | CD8T | TNF is a product of effector CD4 and CD8+ T cells or innate cells | [59] |
|  |  | IFN-gamma | The combination of IFN-γ/GM-CSF was consistently capable of inducing substantial TNF-α mRNA transcript levels and protein secretion | [60] |
|  |  | TNF-a | Autocrine action by TNFα promotes additional TNFα production by macrophages | [61] |
|  |  | ROS | ROS can stimulate the production of TNF-α | [62] |
|  |  | IL-1B | IL-1β were effective when given as a single stimulus for the production of TNF | [63] |
|  |  | IL-10 | IL-10 is a major anti-inflammatory cytokine and functions to inhibit production of TLR-induced proinflammatory mediators, such as TNF | [17] |
|  |  | sTNF-R | Soluble TNF receptors neutralize TNF-α induced cytotoxicity and immunoreactivity in vitro | [64] |
| IL-1B | Interleukin 1 beta | Act-Mon | IL-1β secretion in monocytes is central to the initiation of immune response | [65] |
|  |  | Mac-M1 | These M1 type macrophages produce and secrete higher levels of pro-inflammatory cytokines including IL-1β. | [19] |
|  |  | TNF-a | Autocrine action by TNFα promotes additional production and release of IL-1 | [61] |
|  |  | Act-PLT | Platelet TLR4 induces enhanced splicing and translation of IL-1β | [44] |
|  |  | IL-10 | IL-10 inhibit production of TLR-induced proinflammatory mediators | [17] |
|  |  | IL-1Ra | IL-1Ra is specific for preventing the activity of IL-1α and IL-1β | [9] |
| IFN-gamma | Interferon gamma | Act-NK | Natural killer cells secrete cytokines such as IFN-γ | [66] |
|  |  | Mac-M1 | Human macrophages in vitro through stimulation with IL-12 and IL-18 were able to produce IFN-γ | [67] |
|  |  | CD4T | Naive CD4+ T cells can develop into cells that produced IFN-γ | [68] |
|  |  | CD8T | The ability of CD8+ T cells to produce IFNγ enhanced their ability to migrate to the site of antigen-presenting skin cells. | [69] |
|  |  | Act-DC | Human DCs were most consistently found to stimulate cytotoxicity and IFN-γ secretion of NK cells through IL-12 | [70] |
|  |  | IL-12 | IL-12 is a proinflammatory cytokine that facilitates IFN-γ production by Th1 cells particularly in conjunction with IL-18 | [36] |
|  |  | IL-18 | IL-18 is a proinflammatory cytokine that facilitates IFN-γ production by Th1 cells particularly in conjunction with IL-12 | [36] |
|  |  | IL-6 | Without a positive feedback loop through endogenous IFNγ, CD4+ T cells stimulated in the presence of IL-6 produce less IFNγ | [71] |
|  |  | IL-10 | This endogenous IL-10 reduces TNF, IFNγ, and MIP-2 levels | [72] |
| IL-6 | Interleukin 6 | Mac-M1 | These type 1 macrophages produce and secrete higher levels of IL-6 | [19] |
|  |  | Act-Mon | Activated human peripheral blood monocytes produce IL-6 | [73] |
|  |  | IL-1B | IL-1 induces the local production of Interleukin-6 | [74] |
|  |  | Act-EC | IL-6 is produced also by endothelial cells | [75] |
|  |  | Bcell | B lymphocytes can produce IL-6 | [76] |
|  |  | Act-DC | DC maturation result in secretion of the inflammatory cytokines IL-6 | [57] |
|  |  | IL-10 | IL-10 limits the production of proinflammatory cytokines and chemokines | [72] |
| IL-8 | Interleukin 8 | Act-Mon | Monocytes/macrophages can produce IL-8. | [77] |
|  |  | Mac-M1 | Monocytes/macrophages can produce IL-8. | [77] |
|  |  | Act-EC | Endothelial cells are main producers of Interleukin 8 | [78] |
|  |  | TNF-a | TNFα induced IL-8 expression in human micro vessel endothelial cell line | [79] |
|  |  | IL-10 | IL-10 have a down-regulatory effect on IL-8 secretion | [80] |
| IL-12 | Interleukin 12 | Act-Mon | LPS ablate the IL-12 productive capacity of primary human monocytes | [81] |
|  |  | Act-DC | DC should constitute a critical source of IL-12 | [82] |
|  |  | Mac-M1 | These M1 type macrophages produce and secrete higher levels of IL-12 | [19] |
|  |  | Act-NK | The activated NK cells then prime DC to produce IL-12 | [83] |
|  |  | IFN-gamma | IFN-γ provides a powerful stimulation signal for activated macrophages with a much enhanced potential to produce IL-12 | [84] |
|  |  | IL-1B | IL-1β is identified as a new IL-12-inducing agent on human monocyte-derived DCs | [85] |
|  |  | IL-10 | IL-10 can inhibit IL-12 production in an autocrine manner. | [84] |
| IL-18 | Interleukin 18 | Mac-M1 | Macrophages are the primary sources for the release of active IL-18. | [86] |
|  |  | Act-DC | Dendritic cells are the primary sources for the release of active IL-18. | [86] |
|  |  | Act-EC | Many investigators reported IL-18 production in endothelial cells | [36] |
|  |  | IL-10 | IL-10 limits the production of proinflammatory cytokines and chemokines | [72] |
| IL-10 | Interleukin 10 | Mac-M2 | M2 macrophages have an anti-inflammatory cytokine profile, which characterized by high production of both IL-10 and TGF-β. | [19] |
|  |  | CD4T | IL-10 is expressed by many cells of the adaptive immune system, including TH1, TH2 and TH17 cell subsets, TReg cells, CD8+ T cells and B cells | [87] |
|  |  | CD8T | IL-10 is expressed by many cells of the adaptive immune system, including TH1, TH2 and TH17 cell subsets, TReg cells, CD8+ T cells and B cells | [87] |
|  |  | Treg | IL-10 is expressed by many cells of the adaptive immune system, including TH1, TH2 and TH17 cell subsets, TReg cells, CD8+ T cells and B cells | [87] |
|  |  | Bcell | Autocrine IL-10 promotes human B-cell differentiation and B-cell derived IL-10 | [88] |
|  |  | Act-DC | Il-10 is also expressed by cells of the innate immune system, including DCs | [87] |
|  |  | IL-10 | Autocrine IL-10 increases IL-10 production by DCs | [24] |
|  |  | IL-12 | IL-12 is capable of potently inducing its own inhibitor IL-10 | [89] |
|  |  | Apoptosis | Some apoptotic cells themselves are a potential source of IL-10 | [90] |
|  |  | IFN-gamma | IFN-γ suppresses IL-10 production by increasing the activity of glycogen synthase kinase 3β | [15] |
| sTNF-R | Soluble TNF receptor | Act-Mon | Both the p75 and p55 TNF receptors were measured for blood monocytes incubated in vitro | [91] |
|  |  | CD4T | Soluble TNF receptor production by activated T lymphocytes; p75 sTNF-R production upon stimulation is a feature to all subsets of T cells | [92] |
|  |  | CD8T | p75 sTNF-R production upon stimulation is a feature to all subsets of T cells | [92] |
|  |  | IL-10 | IL-10 increased surface and soluble p75 TNF receptor levels on monocytes | [91] |
| IL-1Ra | Interleukin 1 receptor antagonist | Act-Neu | Human neutrophils produce high levels of the interleukin-1 receptor antagonist | [93] |
|  |  | Act-Mon | Human monocytes secrete an interleukin-1 receptor antagonist | [94] |
|  |  | IFN-gamma | IFN-γ also suppresses the expression of IFN-β- and IL-4-induced IL-1Ra | [95] |
| CD4T | CD4+ T cells | Act-DC | Naïve CD4+ T cells are activated by dendritic cell-derived exosomes | [96] |
|  |  | IL-6 | IL-6 was described as a costimulatory molecule for T cell activation | [97] |
|  |  | IFN-gamma | IFN-γ also upregulates cell surface MHC class II on APCs, thus promoting peptide-specific activation of CD4+ T cells | [98] |
|  |  | IL-12 | IL-12 substantially enhanced the ability of naive CD4+ T cells to develop into cells that produced IFN-γ upon re-stimulation | [68] |
|  |  | Treg | Regulatory T cells control immune activation by acting directly on conventional CD4+ and CD8+ T cells | [23] |
|  |  | IL-10 | IL-10 inhibits the ability of DCs and macrophages to stimulate antigen-specific CD4+ T cells | [99] |
|  |  | Apoptosis | Extensive lymphocyte apoptosis is seen in sepsis | [100] |
| CD8T | CD8+ T cells | IL-18 | IL-18 directly activates CD8+ T cells | [36] |
|  |  | Act-DC | DCs have a strong ability to activate CD8+ cytotoxic T lymphocytes | [22] |
|  |  | Act-NK | The activated NK cells prime DC to induce highly protective CD8+ T cell memory responses | [83] |
|  |  | Treg | Regulatory T-cells control immune activation by acting directly on conventional CD4+ and CD8+ T cells | [23] |
|  |  | IL-10 | IL-10 induces alloantigen-specific unresponsiveness in human CD8+ T cells | [101] |
|  |  | Apoptosis | Extensive lymphocyte apoptosis is seen in sepsis | [100] |
| Treg | Regulatory T cells | CD4T | CD4+ T cells are commonly divided into regulatory T (Treg) cells | [102] |
|  |  | IL-10 | The presence of IL-10 leads to increased expansion of Foxp3+ Tregs. | [103] |
|  |  | IL-6 | IL-6 inhibits TGF-β-induced Treg differentiation | [104] |
|  |  | Apoptosis | Extensive lymphocyte apoptosis is seen in a sepsis | [100] |
| Bcell | B cells | TLR4 | The activation of B cells by microorganisms takes place also through the activation of TLRs | [105] |
|  |  | Act-DC | Antigen presentation by DCs lead to B cell activation | [106] |
|  |  | IL-6 | IL-6 has been shown to induce the final maturation of B cells | [107] |
|  |  | Treg | Tregs can directly suppress B cell response | [108] |
|  |  | IL-10 | IL-10 contribute to the differentiation of IL-10-secreting B cells into IgM- and IgG-secreting plasma blasts | [88] |
|  |  | Apoptosis | Extensive lymphocyte apoptosis is seen in sepsis | [100] |

**References**

1. Bayly-Jones C, Bubeck D, Dunstone MA (2017) The mystery behind membrane insertion: A review of the complement membrane attack complex. Philos. Trans. R. Soc. B Biol. Sci.

2. Uribe-Querol E, Rosales C (2020) Phagocytosis: Our Current Understanding of a Universal Biological Process. Front. Immunol.

3. Dupré-Crochet S, Erard M, Nüβe O (2013) ROS production in phagocytes: why, when, and where? J Leukoc Biol. https://doi.org/10.1189/jlb.1012544

4. Fuchs TA, Abed U, Goosmann C, et al (2007) Novel cell death program leads to neutrophil extracellular traps. J Cell Biol. https://doi.org/10.1083/jcb.200606027

5. Whitfield C, Stephen Trent M (2014) Biosynthesis and export of bacterial lipopolysaccharides. Annu. Rev. Biochem.

6. Lu YC, Yeh WC, Ohashi PS (2008) LPS/TLR4 signal transduction pathway. Cytokine

7. Bosisio D, Polentarutti N, Sironi M, et al (2002) Stimulation of toll-like receptor 4 expression in human mononuclear phagocytes by interferon-γ: A molecular basis for priming and synergism with bacterial lipopolysaccharide. Blood. https://doi.org/10.1182/blood.V99.9.3427

8. Tsujimoto H, Ono S, Hiraki S, et al (2004) Hemoperfusion with polymyxin B-immobilized fibers reduced the number of CD16+CD14 + monocytes in patients with septic shock. J Endotoxin Res. https://doi.org/10.1177/09680519040100040501

9. Dinarello CA (2011) Interleukin-1 in the pathogenesis and treatment of inflammatory diseases. Blood. https://doi.org/10.1182/blood-2010-07-273417

10. Haanen C, Vermes I (1995) Apoptosis and inflammation. Mediators Inflamm 4:5–15. https://doi.org/10.1155/S0962935195000020

11. Tsujimoto H, Ono S, Efron PA, et al (2008) Role of toll-like receptors in the development of sepsis. Shock

12. Shi C, Pamer EG (2011) Monocyte recruitment during infection and inflammation. Nat. Rev. Immunol.

13. Bohlson SS, O’Conner SD, Hulsebus HJ, et al (2014) Complement, C1Q, and C1q-related molecules regulate macrophage polarization. Front. Immunol.

14. Shimaoka M, Park EJ (2008) Advances in understanding sepsis. Eur. J. Anaesthesiol.

15. Hu X, Ivashkiv LB (2009) Cross-regulation of Signaling Pathways by Interferon-γ: Implications for Immune Responses and Autoimmune Diseases. Immunity

16. Schenk M, Fabri M, Krutzik SR, et al (2014) Interleukin-1β triggers the differentiation of macrophages with enhanced capacity to present mycobacterial antigen to T cells. Immunology. https://doi.org/10.1111/imm.12167

17. Fiorentino DF, Zlotnik A, Mosmann TR, et al (1991) IL-10 inhibits cytokine production by activated macrophages. J Immunol

18. Murray PJ, Wynn TA (2011) Protective and pathogenic functions of macrophage subsets. Nat. Rev. Immunol.

19. Shapouri-Moghaddam A, Mohammadian S, Vazini H, et al (2018) Macrophage plasticity, polarization, and function in health and disease. J. Cell. Physiol.

20. Wang N, Liang H, Zen K (2014) Molecular mechanisms that influence the macrophage M1-M2 polarization balance. Front. Immunol.

21. Luz-Crawford P, Djouad F, Toupet K, et al (2016) Mesenchymal Stem Cell-Derived Interleukin 1 Receptor Antagonist Promotes Macrophage Polarization and Inhibits B Cell Differentiation. Stem Cells. https://doi.org/10.1002/stem.2254

22. Fang H, Ang B, Xu X, et al (2014) TLR4 is essential for dendritic cell activation and anti-tumor T-cell response enhancement by DAMPs released from chemically stressed cancer cells. Cell Mol Immunol. https://doi.org/10.1038/cmi.2013.59

23. Rueda CM, Jackson CM, Chougnet CA (2016) Regulatory T-cell-mediated suppression of conventional T-cells and dendritic cells by different cAMP intracellular pathways. Front. Immunol.

24. Corinti S, Albanesi C, la Sala A, et al (2001) Regulatory Activity of Autocrine IL-10 on Dendritic Cell Functions. J Immunol. https://doi.org/10.4049/jimmunol.166.7.4312

25. Sabroe I, Prince LR, Jones EC, et al (2003) Selective Roles for Toll-Like Receptor (TLR)2 and TLR4 in the Regulation of Neutrophil Activation and Life Span. J Immunol. https://doi.org/10.4049/jimmunol.170.10.5268

26. Denk S, Taylor RP, Wiegner R, et al (2017) Complement C5a-Induced Changes in Neutrophil Morphology During Inflammation. Scand J Immunol. https://doi.org/10.1111/sji.12580

27. Zeilhofer HU, Schorr W (2000) Role of interleukin-8 in neutrophil signaling. Curr. Opin. Hematol.

28. Naume B, Gately M, Espevik T (1992) A comparative study of IL-12 (cytotoxic lymphocyte maturation factor)-, IL-2-, and IL-7-induced effects on immunomagnetically purified CD56+ NK cells. J Immunol

29. Fehniger TA, Shah MH, Turner MJ, et al (1999) Differential cytokine and chemokine gene expression by human NK cells following activation with IL-18 or IL-15 in combination with IL-12: implications for the innate immune response. J Immunol

30. Ferlazzo G, Tsang ML, Moretta L, et al (2002) Human dendritic cells activate resting natural killer (NK) cells and are recognized via the NKp30 receptor by activated NK cells. J Exp Med. https://doi.org/10.1084/jem.20011149

31. Folco EJ, Mawson TL, Vromman A, et al (2018) Neutrophil extracellular traps induce endothelial cell activation and tissue factor production through interleukin-1α and cathepsin G. Arterioscler Thromb Vasc Biol. https://doi.org/10.1161/ATVBAHA.118.311150

32. Pober JS, Sessa WC (2007) Evolving functions of endothelial cells in inflammation. Nat. Rev. Immunol.

33. Egholm C, Heeb LEM, Impellizzieri D, Boyman O (2019) The regulatory effects of interleukin-4 receptor signaling on neutrophils in type 2 immune responses. Front. Immunol.

34. Heesterbeek DAC, Angelier ML, Harrison RA, Rooijakkers SHM (2018) Complement and Bacterial Infections: From Molecular Mechanisms to Therapeutic Applications. J. Innate Immun.

35. Drevets DA, Leenen PJM, Campbell PA (1996) Complement receptor type 3 mediates phagocytosis and killing of Listeria monocytogenes by a TNF-α- and IFN-γ-stimulated macrophage precursor hybrid. Cell Immunol. https://doi.org/10.1006/cimm.1996.0083

36. Yasuda K, Nakanishi K, Tsutsui H (2019) Interleukin-18 in health and disease. Int. J. Mol. Sci.

37. Rath PC, Aggarwal BB (1999) TNF-induced signaling in apoptosis. J. Clin. Immunol.

38. Eeva J, Pelkonen J (2004) Mechanisms of B cell receptor induced apoptosis. Apoptosis

39. Nolan Y, Vereker E, Lynch AM, Lynch MA (2003) Evidence that lipopolysaccharide-induced cell death is mediated by accumulation of reactive oxygen species and activation of p38 in rat cortex and hippocampus. Exp Neurol. https://doi.org/10.1016/S0014-4886(03)00301-7

40. Silva M, Videira PA, Sackstein R (2018) E-selectin ligands in the human mononuclear phagocyte system: Implications for infection, inflammation, and immunotherapy. Front. Immunol.

41. Rahman A, Kefer J, Bando M, et al (1998) E-selectin expression in human endothelial cells by TNF-α-induced oxidant generation and NF-κB activation. Am J Physiol - Lung Cell Mol Physiol. https://doi.org/10.1152/ajplung.1998.275.3.l533

42. Nguyen GT, Green ER, Mecsas J (2017) Neutrophils to the ROScue: Mechanisms of NADPH oxidase activation and bacterial resistance. Front. Cell. Infect. Microbiol.

43. Carestia A, Kaufman T, Schattner M (2016) Platelets: New bricks in the building of neutrophil extracellular traps. Front. Immunol.

44. Herter JM, Rossaint J, Zarbock A (2014) Platelets in inflammation and immunity. J. Thromb. Haemost.

45. Yun SH, Sim EH, Goh RY, et al (2016) Platelet activation: The mechanisms and potential biomarkers. Biomed Res. Int.

46. Stokes KY, Granger DN (2012) Platelets: A critical link between inflammation and microvascular dysfunction. J. Physiol.

47. Franco RF, De Jonge E, Dekkers PEP, et al (2000) The in vivo kinetics of tissue factor messenger RNA expression during human endotoxemia: Relationship with activation of coagulation. Blood. https://doi.org/10.1182/blood.v96.2.554

48. Witkowski M, Landmesser U, Rauch U (2016) Tissue factor as a link between inflammation and coagulation. Trends Cardiovasc. Med.

49. Manly DA, Boles J, MacKman N (2011) Role of tissue factor in venous thrombosis. Annu Rev Physiol. https://doi.org/10.1146/annurev-physiol-042210-121137

50. Rooijakkers SHM, van Strijp JAG (2007) Bacterial complement evasion. Mol. Immunol.

51. Noris M, Remuzzi G (2013) Overview of complement activation and regulation. Semin Nephrol. https://doi.org/10.1016/j.semnephrol.2013.08.001

52. Canton M, Sánchez-Rodríguez R, Spera I, et al (2021) Reactive Oxygen Species in Macrophages: Sources and Targets. Front. Immunol.

53. Montezano AC, Touyz RM (2012) Reactive oxygen species and endothelial function - Role of nitric oxide synthase uncoupling and nox family nicotinamide adenine dinucleotide phosphate oxidases. Basic Clin. Pharmacol. Toxicol.

54. Kim JJ, Lee SB, Park JK, Yoo YD (2010) TNF-α-induced ROS production triggering apoptosis is directly linked to Romo1 and Bcl-XL. Cell Death Differ. https://doi.org/10.1038/cdd.2010.19

55. Hegen A, Koidl S, Weindel K, et al (2004) Expression of angiopoietin-2 in endothelial cells is controlled by positive and negative regulatory promoter elements. Arterioscler Thromb Vasc Biol. https://doi.org/10.1161/01.ATV.0000140819.81839.0e

56. Deree J, Martins JO, Melbostad H, et al (2008) Insights into the regulation of TNF-α production in human mononuclear cells: The effects of non-specific phosphodiesterase inhibition. Clinics. https://doi.org/10.1590/S1807-59322008000300006

57. Reddy ST, Swartz MA, Hubbell JA (2006) Targeting dendritic cells with biomaterials: developing the next generation of vaccines. Trends Immunol.

58. Paul S, Lal G (2017) The molecular mechanism of natural killer cells function and its importance in cancer immunotherapy. Front. Immunol.

59. Mehta AK, Gracias DT, Croft M (2018) TNF activity and T cells. Cytokine. https://doi.org/10.1016/j.cyto.2016.08.003

60. Kohn FR, Phillips GL, Klingemann HG (1992) Regulation of tumor necrosis factor-α production and gene expression in monocytes. Bone Marrow Transplant

61. Ott LW, Resing KA, Sizemore AW, et al (2007) Tumor necrosis factor-α- and interleukin-1-induced cellular responses: Coupling proteomic and genomic information. J Proteome Res. https://doi.org/10.1021/pr060665l

62. Naik E, Dixit VM (2011) Mitochondrial reactive oxygen species drive proinflammatory cytokine production. J. Exp. Med.

63. Hoffmann G, Schloesser M, Czechowski M, et al (2004) Tumor necrosis factor-α gene expression and release in cultured human dermal microvascular endothelial cells. Exp Dermatol. https://doi.org/10.1111/j.0906-6705.2004.00142.x

64. Van Zee KJ, Kohno T, Fischer E, et al (1992) Tumor necrosis factor soluble receptors circulate during experimental and clinical inflammation and can protect against excessive tumor necrosis factor α in vitro and in vivo. Proc Natl Acad Sci U S A. https://doi.org/10.1073/pnas.89.11.4845

65. Hadadi E, Zhang B, Baidzajevas K, et al (2016) Differential IL-1β secretion by monocyte subsets is regulated by Hsp27 through modulating mRNA stability. Sci Rep. https://doi.org/10.1038/srep39035

66. Girart M V., Fuertes MB, Domaica CI, et al (2007) Engagement of TLR3, TLR7, and NKG2D Regulate IFN-γ Secretion but Not NKG2D-Mediated Cytotoxicity by Human NK Cells Stimulated with Suboptimal Doses of IL-12. J Immunol. https://doi.org/10.4049/jimmunol.179.6.3472

67. Darwich L, Coma G, Peña R, et al (2009) Secretion of interferon-γ by human macrophages demonstrated at the single-cell level after costimulation with interleukin (IL)-12 plus IL-18. Immunology. https://doi.org/10.1111/j.1365-2567.2008.02905.x

68. Seder RA, Gazzinelli R, Sher A, Paul WE (1993) Interleukin 12 acts directly on CD4+ T cells to enhance priming for interferon γ production and diminishes interleukin 4 inhibition of such priming. Proc Natl Acad Sci U S A. https://doi.org/10.1073/pnas.90.21.10188

69. Bhat P, Leggatt G, Waterhouse N, Frazer IH (2017) Interferon-γ derived from cytotoxic lymphocytes directly enhances their motility and cytotoxicity. Cell Death Dis. https://doi.org/10.1038/cddis.2017.67

70. Ferlazzo G, Pack M, Thomas D, et al (2004) Distinct roles of IL-12 and IL-15 in human natural killer cell activation by dendritic cells from secondary lymphoid organs. Proc Natl Acad Sci U S A. https://doi.org/10.1073/pnas.0407522101

71. Dienz O, Rincon M (2009) The effects of IL-6 on CD4 T cell responses. Clin. Immunol.

72. Moore KW, De Waal Malefyt R, Coffman RL, O’Garra A (2001) Interleukin-10 and the interleukin-10 receptor. Annu. Rev. Immunol.

73. Vellenga E, Tuyt L, Wierenga BJ, et al (1999) Interleukin-6 production by activated human monocytic cells is enhanced by MK-571, a specific inhibitor of the multi-drug resistance protein-1. Br J Pharmacol. https://doi.org/10.1038/sj.bjp.0702577

74. Tosato G, Jones KD (1990) Interleukin-1 induces interleukin-6 production in peripheral blood monocytes. Blood. https://doi.org/10.1182/blood.v75.6.1305.1305

75. Kishimoto T (2005) Interleukin-6: From basic science to medicine - 40 Years in immunology. Annu. Rev. Immunol.

76. Kawano M, Hirano T, Matsuda T, et al (1988) Autocrine generation and requirement of BSF-2/IL-6 for human multiple myelomas. Nature. https://doi.org/10.1038/332083a0

77. Jundi K, Greene CM (2015) Transcription of interleukin-8: How altered regulation can affect cystic fibrosis lung disease. Biomolecules

78. Nijhuis CSMO, Vellenga E, Daenen SMGJ, et al (2003) Endothelial cells are main producers of interleukin 8 through toll-like receptor 2 and 4 signaling during bacterial infection in leukopenic cancer patients. Clin Diagn Lab Immunol. https://doi.org/10.1128/CDLI.10.4.558-563.2003

79. Lakshminarayanan V, Beno DWA, Costa RH, Roebuck KA (1997) Differential regulation of interleukin-8 and intercellular adhesion molecule-1 by H2O2 and tumor necrosis factor-α in endothelial and epithelial cells. J Biol Chem. https://doi.org/10.1074/jbc.272.52.32910

80. Ameixa C, Friedland JS (2001) Down-regulation of interleukin-8 secretion from Mycobacterium tuberculosis-infected monocytes by interleukin-4 and -10 but not by interleukin-13. Infect Immun. https://doi.org/10.1128/IAI.69.4.2470-2476.2001

81. Karp CL, Wysocka M, Ma X, et al (1998) Potent suppression of IL-12 production from monocytes and dendritic cells during endotoxin tolerance. Eur J Immunol. https://doi.org/10.1002/(SICI)1521-4141(199810)28:10<3128::AID-IMMU3128>3.0.CO;2-T

82. Heufler C, Koch F, Stanzl U, et al (1996) Interleukin-12 is produced by dendritic cells and mediates T helper 1 development as well as interferon-γ production by T helper 1 cells. Eur J Immunol. https://doi.org/10.1002/eji.1830260323

83. Mocikat R, Braumüller H, Gumy A, et al (2003) Natural killer cells activated by MHC class ILow targets prime dendritic cells to induce protective CD8 T cell responses. Immunity. https://doi.org/10.1016/S1074-7613(03)00264-4

84. Liu J, Cao S, Kim S, et al (2005) Interleukin-12: An Update on its Immunological Activities, Signaling and Regulation of Gene Expression. Curr Immunol Rev. https://doi.org/10.2174/1573395054065115

85. Wesa AK, Galy A (2001) IL-1β induces dendritic cells to produce IL-12. Int Immunol. https://doi.org/10.1093/intimm/13.8.1053

86. Dinarello CA, Novick D, Kim S, Kaplanski G (2013) Interleukin-18 and IL-18 binding protein. Front. Immunol.

87. Saraiva M, O’Garra A (2010) The regulation of IL-10 production by immune cells. Nat. Rev. Immunol.

88. Heine G, Drozdenko G, Grün JR, et al (2014) Autocrine IL-10 promotes human B-cell differentiation into IgM- or IgG-secreting plasmablasts. Eur J Immunol. https://doi.org/10.1002/eji.201343822

89. Meyaard L, Hovenkamp E, Otto SA, Miedema F (1996) IL-12-induced IL-10 production by human T cells as a negative feedback for IL-12-induced immune responses. J Immunol

90. Albert ML (2004) Death-defying immunity: Do apoptotic cells influence antigen processing and presentation? Nat. Rev. Immunol.

91. Hart PH, Hunt EK, Bonder CS, et al (1996) Regulation of surface and soluble TNF receptor expression on human monocytes and synovial fluid macrophages by IL-4 and IL-10. J Immunol

92. Cope AP, Aderka D, Wallach D, et al (1995) Soluble TNF receptor production by activated T lymphocytes: differential effects of acute and chronic exposure to TNF. Immunology

93. McColl SR, Paquin R, Ménard C, Beaulieu AD (1992) Human neutrophils produce high levels of the interleukln 1 receptor antagonist in response to granulocyte/macrophage colony-stimulating factor and tumor necrosis factor α. J Exp Med. https://doi.org/10.1084/jem.176.2.593

94. Eisenberg SP, Evans RJ, Arend WP, et al (1990) Primary structure and functional expression from complementary DNA of a human interleukin-1 receptor antagonist. Nature. https://doi.org/10.1038/343341a0

95. Sone S, Orino E, Mizuno K, et al (1994) Production of IL-1 and its receptor antagonist is regulated differently by IFN-γ and IL-4 in human monocytes and alveolar macrophages. Eur Respir J. https://doi.org/10.1183/09031936.94.07040657

96. Théry C, Duban L, Segura E, et al (2002) Indirect activation of naïve CD4+ T cells by dendritic cell-derived exosomes. Nat Immunol. https://doi.org/10.1038/ni854

97. Lotz M, Jirik F, Kabouridis P, et al (1988) B cell stimulating factor 2/interleukin 6 is a costimulant for human thymocytes and T lymphocytes. J Exp Med. https://doi.org/10.1084/jem.167.3.1253

98. Castro F, Cardoso AP, Gonçalves RM, et al (2018) Interferon-gamma at the crossroads of tumor immune surveillance or evasion. Front. Immunol.

99. Mittal SK, Cho KJ, Ishido S, Roche PA (2015) Interleukin 10 (IL-10)-mediated Immunosuppression March-i induction regulates antigen presentation by macrophages but not Dendritic cells. J Biol Chem. https://doi.org/10.1074/jbc.M115.682708

100. De Pablo R, Monserrat J, Prieto A, Alvarez-Mon M (2014) Role of Circulating Lymphocytes in Patients with Sepsis. Biomed Res. Int.

101. Groux H, Bigler M, de Vries JE, Roncarolo MG (1998) Inhibitory and stimulatory effects of IL-10 on human CD8+ T cells. J Immunol

102. Corthay A (2009) How do regulatory t cells work? Scand. J. Immunol.

103. Hsu P, Santner-Nanan B, Hu M, et al (2015) IL-10 Potentiates Differentiation of Human Induced Regulatory T Cells via STAT3 and Foxo1. J Immunol. https://doi.org/10.4049/jimmunol.1402898

104. Kimura A, Kishimoto T (2010) IL-6: Regulator of Treg/Th17 balance. Eur. J. Immunol.

105. Rawlings DJ, Schwartz MA, Jackson SW, Meyer-Bahlburg A (2012) Integration of B cell responses through Toll-like receptors and antigen receptors. Nat. Rev. Immunol.

106. Heath WR, Kato Y, Steiner TM, Caminschi I (2019) Antigen presentation by dendritic cells for B cell activation. Curr. Opin. Immunol.

107. Hirano T, Yasukawa K, Harada H, et al (1986) Complementary DNA for a novel human interleukin (BSF-2) that induces B lymphocytes to produce immunoglobulin. Nature. https://doi.org/10.1038/324073a0

108. Lim HW, Hillsamer P, Banham AH, Kim CH (2005) Cutting Edge: Direct Suppression of B Cells by CD4 + CD25 + Regulatory T Cells . J Immunol. https://doi.org/10.4049/jimmunol.175.7.4180
