# Supplementary figures and images for: A system pharmacology Boolean network model for the TLR4-mediated inflammatory response in early sepsis
Source: J Pharmacokinet Pharmacodyn. 2022 Oct 19;49(6):645–55. doi: 10.1007/s10928-022-09828-6 (PMC9649476; doi:10.1007/s10928-022-09828-6)

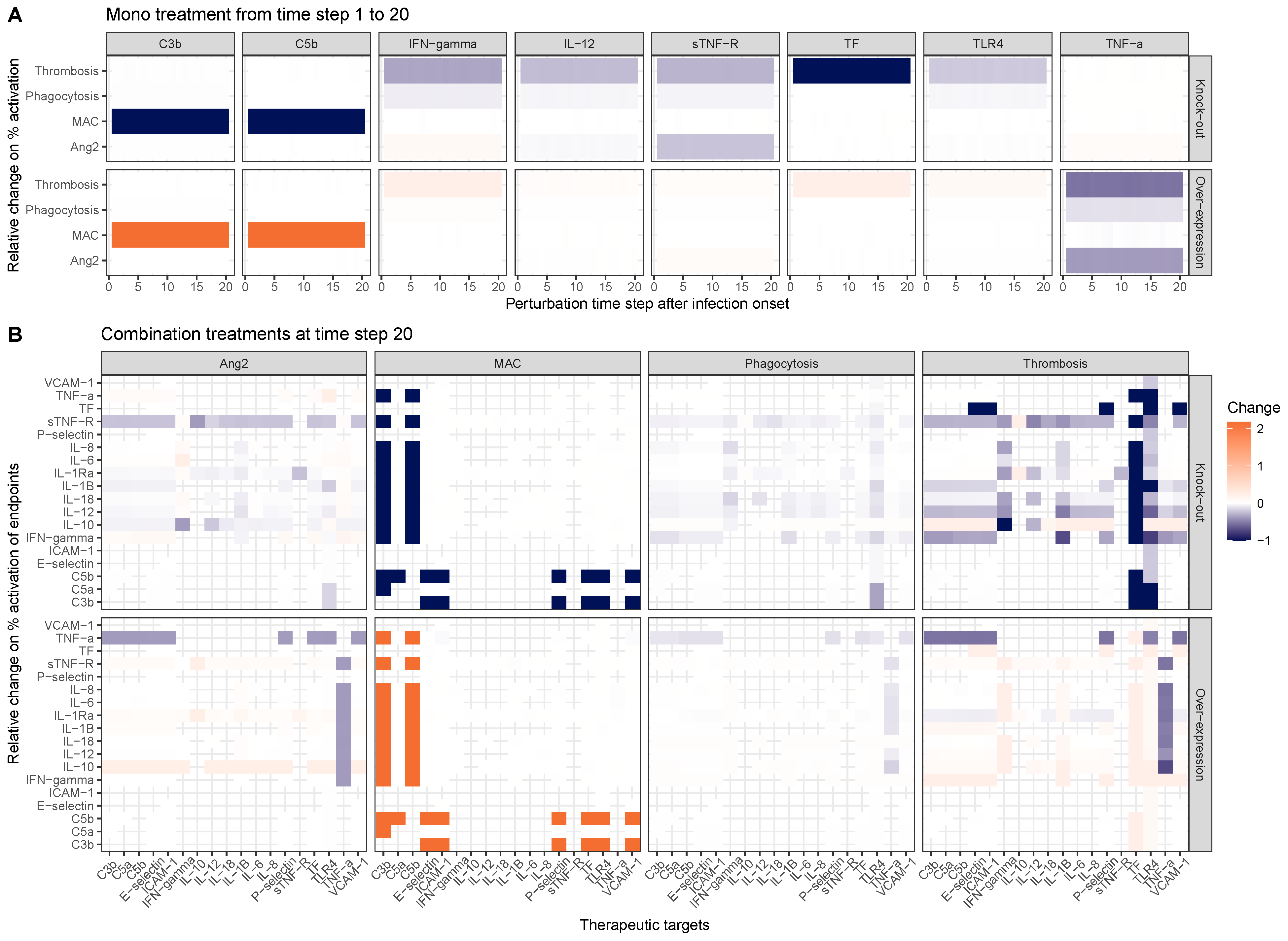

Supplement: Supplementary file 2 — Supplementary material 2 (TIF 2038.8 kb) Fig. S1 Relative changes of four selected endpoints activation under mono and combined perturbations on mediatory molecules. Upper heatmap (A) showed the effect of knocking-out or over-expressing of identified mono therapeutic targets on four endpoints over different perturbation initiation time steps; below heatmaps (B) showed the example of effect of knocking-out or over-expressing combined therapeutic targets on four endpoints when initiating perturbations at time step 20. Colors of the heatmap represented the negative, neutral and positive relative changes of endpoints activation with blue, white and orange, respectively [file 10928_2022_9828_MOESM2_ESM.tif]

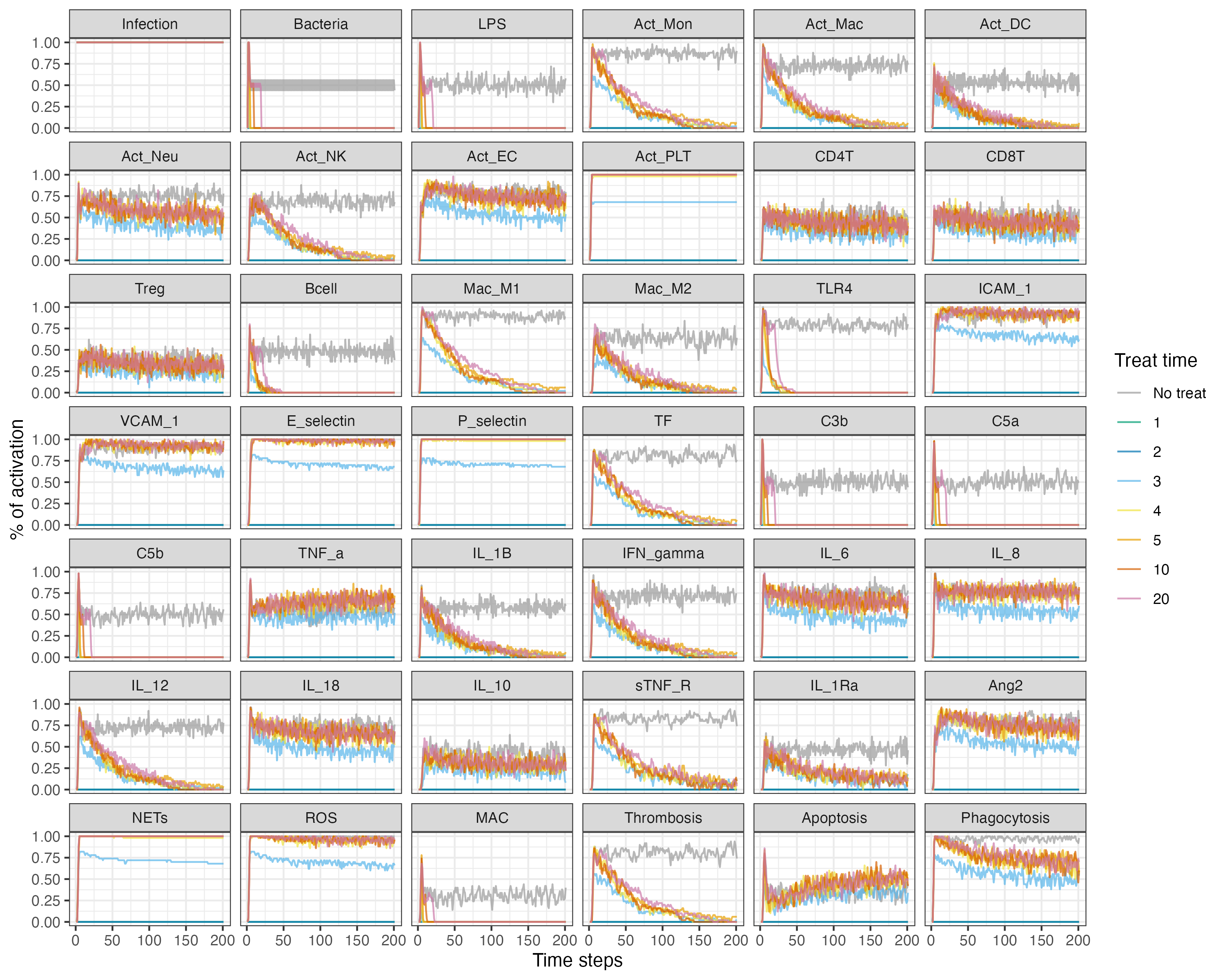

Supplement: Supplementary file 3 — Supplementary material 3 (TIFF 2204.7 kb) Fig. S2 Average activation profiles for each node under antibiotic treatment (i.e. knocking out node Bacteria) at different time step with 100 repetitions. When removing bacteria at an early phase (before time step 4), most nodes were not activated or returned back to baseline immediately; when removing bacteria at a later phase, it showed varying decline patterns for different nodes. Colors of the lines represented different perturbation initiation time steps [file 10928_2022_9828_MOESM3_ESM.tiff]

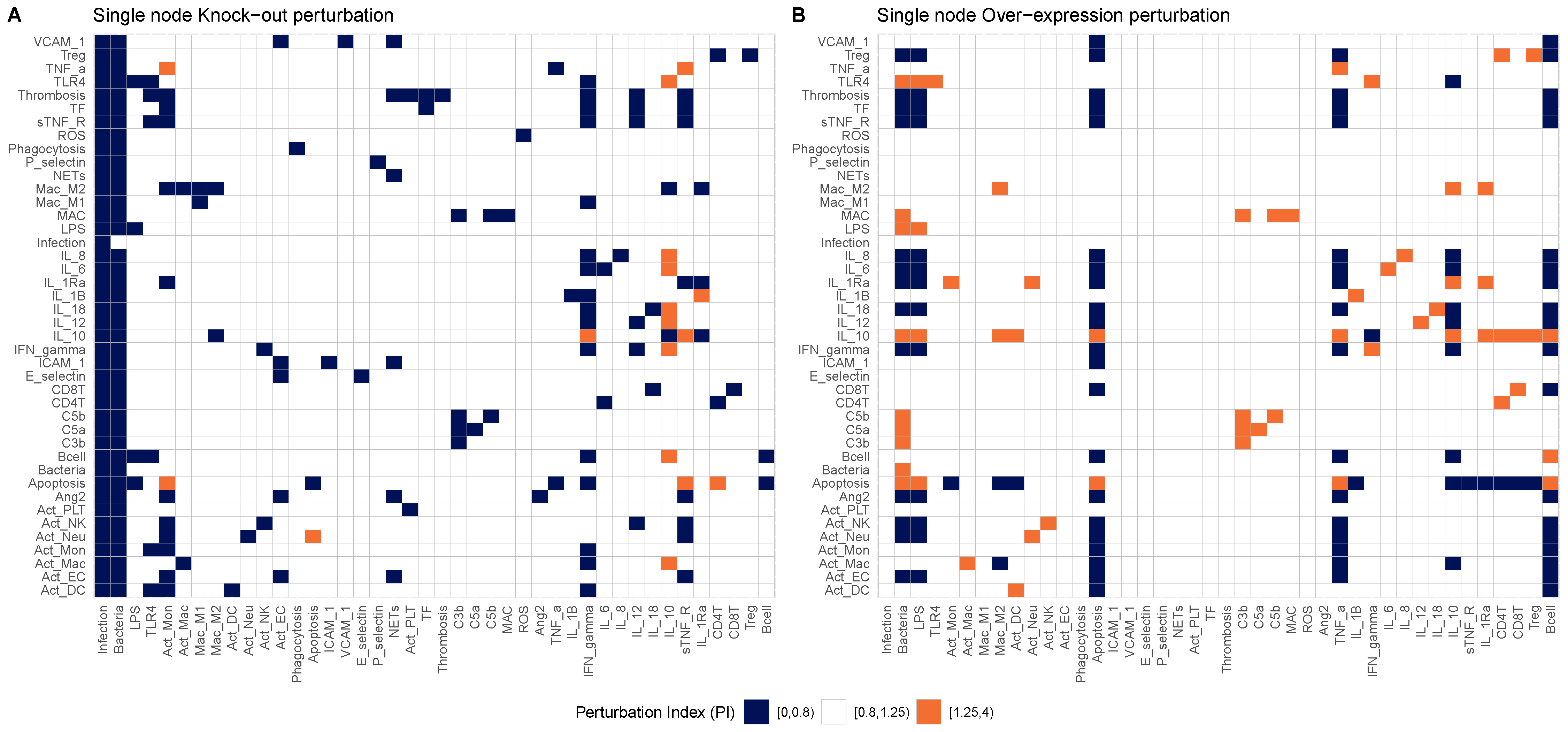

Supplement: Supplementary file 4 — Supplementary material 4 (TIF 928.5 kb) Fig. S3 Overview of single node perturbation analysis of the network. The heatmaps indicated the effect of entire knock-out (A) or over-expression (B) of each node (columns) in every network node (rows). Colors of the heatmap represented the Perturbation Index (PI) with the negative (PI < 0.8), neutral (0.8 < PI < 1.25) and positive (PI > 1.25) changes being blue, white and orange, respectively [file 10928_2022_9828_MOESM4_ESM.tif]

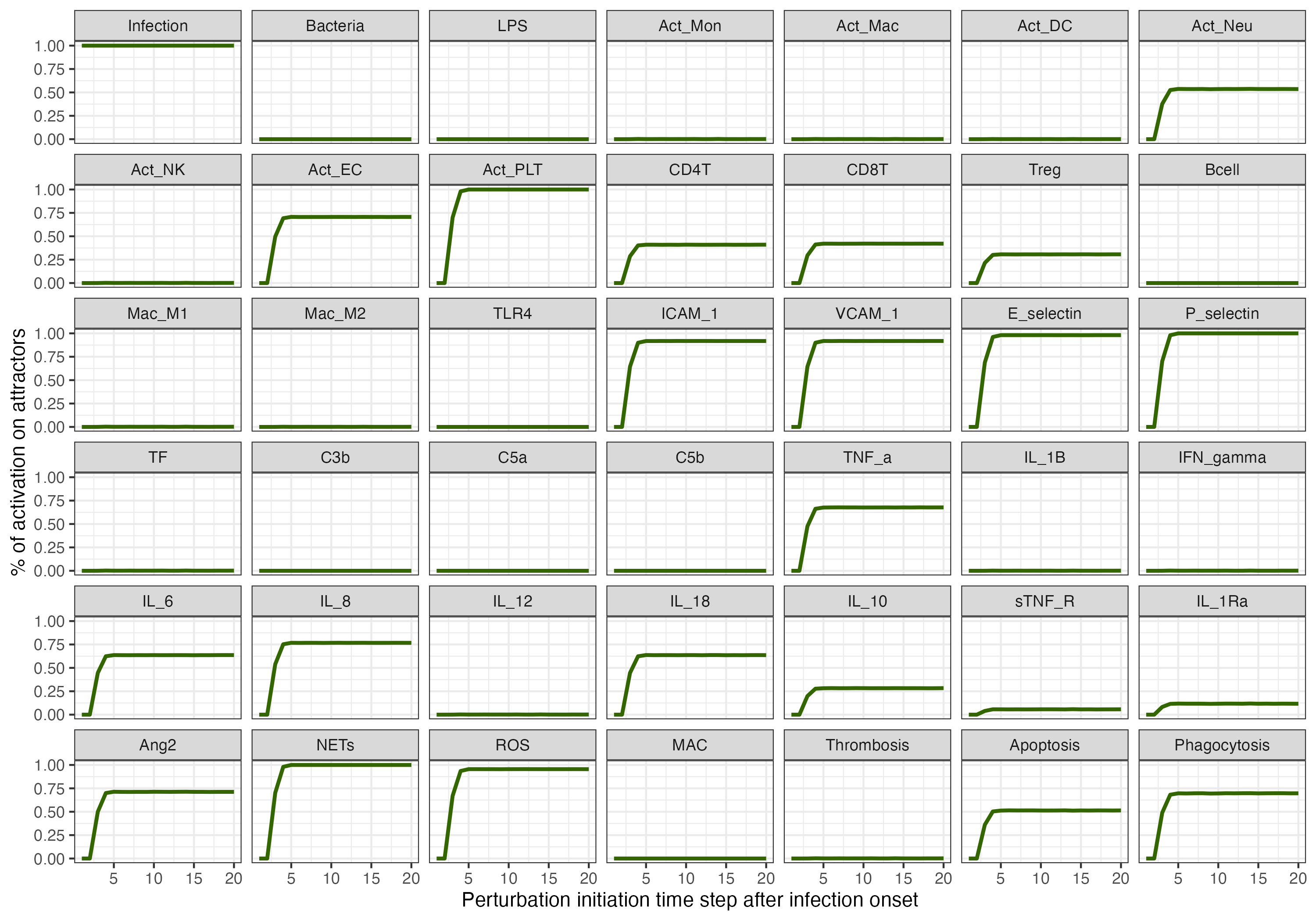

Supplement: Supplementary file 5 — Supplementary material 5 (TIFF 756.0 kb) Fig. S4 Activation of each node on attractors under antibiotic treatment (i.e. knocking out node Bacteria) at different time steps. The activations on attractors stayed unchanged when removing bacteria at a later phase (i.e. after time step 4) [file 10928_2022_9828_MOESM5_ESM.tiff]
